# Supplementary material for: Ganoaustralins A and B, Unusual Aromatic Triterpenes from the Mushroom Ganoderma australe
Source: Pharmaceuticals (Basel). 2022 Dec 6;15(12):1520. doi: 10.3390/ph15121520 (PMC9785556; doi:10.3390/ph15121520)
Supplement: Supplementary file 1 [file pharmaceuticals-15-01520-s001.zip › pharmaceuticals-2068872-supplementary.pdf]

Supplementary Materials for

Ganoaustralins A and B, Unusual Aromatic Triterpenes from the Mushroom *Ganoderma australe*

Lin Zhou[1,†], He-Ping Chen[1,†], Xinyang Li[1,2,*], Ji-Kai Liu[1,*]

[1]School of Pharmaceutical Sciences, South-Central Minzu University, Wuhan 430074, People's Republic of China

[2]Graduate School of Pharmaceutical Sciences, The University of Tokyo, Bunkyo-ku, Tokyo 113-0033, Japan

[†]These authors contribute equally to this work.

*Correspondence: xinyangli131@hotmail.com (X. Li);

jkliu@mail.kib.ac.cn; liujikai@mail.scuec.edu.cn (J.-K. Liu).

Contents

1. Table S1 Triterpenoid scaffolds from fungi. 3
2. Supplementary figures. 6
Figure S1. ^{1}H NMR spectrum of **1** (600 MHz, C_5D_5N). 6
Figure S2. ^{13}C and DEPT NMR spectra of **1** (150 MHz, C_5D_5N). 7
Figure S3. Enlarged ^{13}C and DEPT NMR spectra of **1** (150 MHz, C_5D_5N). 8
Figure S4. HSQC spectrum of **1** (C_5D_5N). 9
Figure S5. ^{1}H-^{1}H COSY spectrum of **1** (C_5D_5N). 10
Figure S6. HMBC spectrum of **1** (C_5D_5N). 11
Figure S7. Enlarged HMBC spectrum A of **1** (C_5D_5N). 12
Figure S8. Enlarged HMBC spectrum B of **1** (C_5D_5N). 13
Figure S9. ROESY spectrum of **1** (C_5D_5N). 14
Figure S10. ^{1}H NMR spectrum of **1** (600 MHz, $CDCl_3$). 15
Figure S11. ^{13}C and DEPT NMR spectra of **1** (150 MHz, $CDCl_3$). 16
Figure S12. Enlarged ^{13}C and DEPT NMR spectra of **1** (150 MHz, $CDCl_3$). 17
Figure S13. HSQC spectrum of **1** ($CDCl_3$). 18
Figure S14. ^{1}H-^{1}H COSY spectrum of **1** ($CDCl_3$). 19
Figure S15. HMBC spectrum of **1** ($CDCl_3$). 20
Figure S16. Enlarged HMBC spectrum A of **1** ($CDCl_3$). 21
Figure S17. ROESY spectrum of **1** ($CDCl_3$). 22
Figure S18. HRESIMS report of **1**. 23
Figure S19. ^{1}H NMR spectrum of **2** (600 MHz, $CDCl_3$). 24
Figure S20. ^{13}C and DEPT135 NMR spectra of **2** (150 MHz, $CDCl_3$). 25
Figure S21. Enlarged ^{13}C and DEPT135 NMR spectra of **2** (150 MHz, $CDCl_3$). 26
Figure S22. HSQC spectrum of **2**. 27
Figure S23. ^{1}H-^{1}H COSY spectrum of **2**. 28
Figure S24. HMBC spectrum of **2**. 29
Figure S25. Enlarged HMBC spectrum A of **2**. 30
Figure S26. Enlarged HMBC spectrum B of **2**. 31
Figure S27. ROESY spectrum of **2**. 32
Figure S28. HRESIMS report of **2**. 33
Figure S29. ^{1}H NMR spectrum of (*S*)-PGME-**1** (600 MHz, $CDCl_3$). 34
Figure S30. ^{1}H NMR spectrum of (*R*)-PGME-**1** (**1a**) (600 MHz, $CDCl_3$). 35
Figure S31. Comparison of ^{1}H NMR spectra of (*S*)- and (*R*)-PGME-**1** (**1a**) (600 MHz, $CDCl_3$). 36
Figure S32. Enlarged comparison of ^{1}H NMR spectra of (*S*)- and (*R*)-PGME-**1** (**1a**) (600 MHz, $CDCl_3$). 37
Figure S33. ^{1}H-^{1}H COSY spectrum of (*S*)-PGME-**1** (**1a**). 38
Figure S34. ^{1}H-^{1}H COSY spectrum of (*R*)-PGME-**1** (**1a**). 39
Figure S35. HRESIMS report of (*S*)-PGME-**1** (**1a**). 40
Figure S36. HRESIMS report of (*R*)-PGME-**1** (**1b**). 41
3. Calculation details. 42
Table S2. Conformational analysis of the M06-2X-D3/Def2-SVP optimized conformers of **1**. 42
Table S3. Calculated ^{13}C NMR analysis of **1**. 42
Table S4. Calculated ^{1}H NMR analysis of **1**. 43
Table S5. Conformational analysis of the M06-2X-D3/Def2-SVP optimized conformers of **2**. 43

Table S6. Calculated ^{13}C NMR analysis of **2**. 44
Table S7. Calculated ^{1}H NMR analysis of **2**. 45
Table S8. Standard orientations of conformers. 46
4. Biological assays 52
Table S9. Cytotoxicity activities of compounds **1** and **2**. 52
Table S10 Inhibition rates of compounds **1** and **2** against BACE1 52
Table S11 Inhibition rates of compounds **1** and **2** against α-glucosidase 52
Table S12 Inhibition rates of compounds **1** and **2** against PTP1B 52

1. Table S1 Triterpenoid scaffolds from fungi.

Scaffold Names[a]	Scaffold Structures	Example	Species & Ref.
Triterpenoid scaffold from Ganoderma			
Lanostane	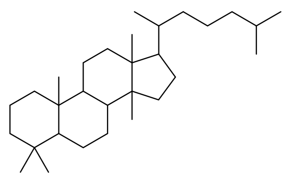	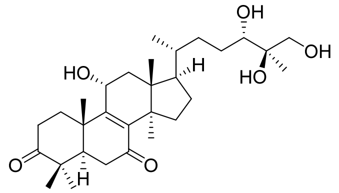 Leucocontextin S	*G. luecocontextum* *Nat. Prod. Bioprospect.* **2016**, 6, 103-109 10.1007/s13659-016-0089-3
14(13→12)*abeo*-lanostane	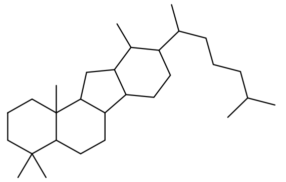	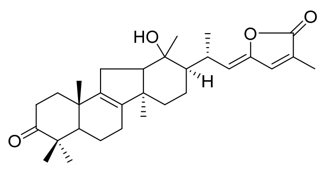 (22Z,24Z)-13-hydroxy-3-oxo-14(13→12)*abeo*-lanosta-8,22,24-trien-26,23-olide	*G. lucidum* *J. Asian Nat. Prod. Res.*, **2015**, 17, 750-755 10.1080/10286020.2014.996139
3,4-*seco*-27-norlanostane	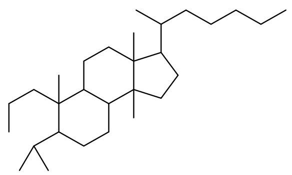	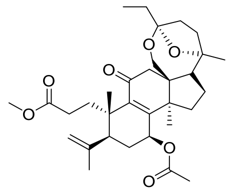 Ganoboninketal A	*G. boninense* *J. Nat. Prod.* **2014**, 77, 1847–1852 10.1021/np5002863
3,4-*seco*-9(10→19)*abeo*-27-norlanostane	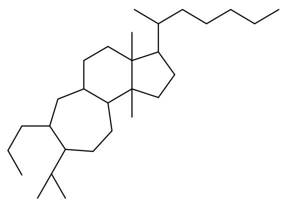	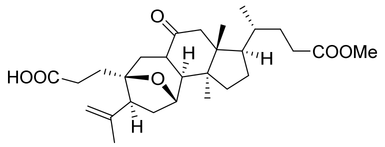 Cochlate A	*G. cochlear* *RSC Adv.*, **2015**,5, 95212-95222 10.1039/C5RA16796E
N.A.	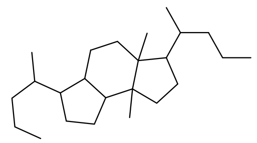	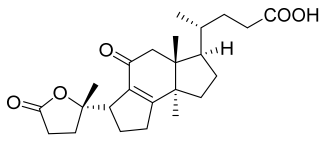 Ganocochlearic acid A	*G. cochlear* *RSC Adv.*, **2015**,5, 95212-95222 10.1039/C5RA16796E
1,11-*cyclo*-27-norlanostane	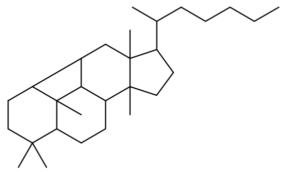	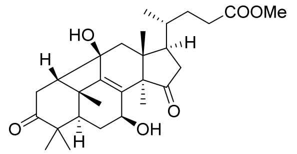 Methyl ganosinensate A	*G. sinense* *Org. Lett.*, **2010**, 8, 1656-1659 10.1021/ol100062b

3,4-*seco*-12,23-*cyclo*-27-norlanostane	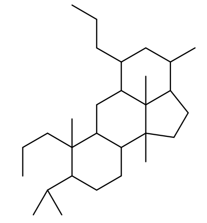	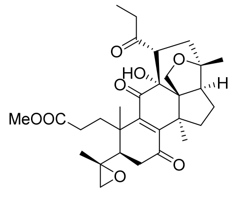 Ganorbifate A	*G. orbiforme*<br>*Chem. Commun.*<br>**2020**,56, 10195-10198<br>10.1039/D0CC04679E
3,4-*seco*-1,11-*cyclo*-27-norlanostane	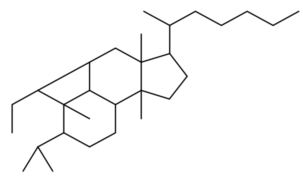	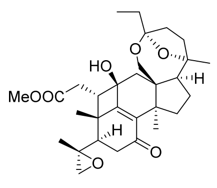 Ganorbifate B	*G. orbiforme*<br>*Chem. Commun.*<br>**2020**,56, 10195-10198<br>10.1039/D0CC04679E
Triterpenoids from other fungal genera, e.g. *Irpex*, *Poria*, *Antrodia*, *Fomes*, *Inonotus*, *Tricholoma*			
irpexonane	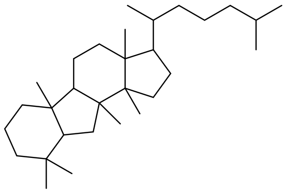	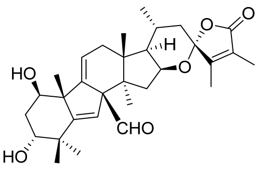 irpexolidal	*Irpex lacteus*<br>*J. Org. Chem.* **2019**, 84, 1845−1852<br>10.1021/acs.joc.8b02764
Eburicane (24-methyllanostane)	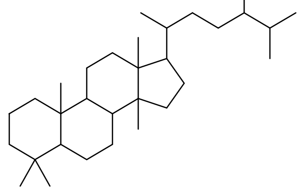	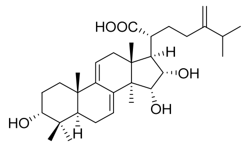 Irpeksolactin B	*Irpex lacteus*<br>*Phytochemistry*, **2019**, 162, 21-28<br>10.1016/j.phytochem.2019.02.017
4,5-*seco*-19(10→5)-eburicane	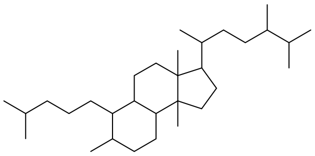	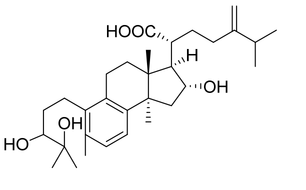 Irpeksolactin A	*Irpex lacteus*<br>*Phytochemistry*, **2019**, 162, 21-28<br>10.1016/j.phytochem.2019.02.017
7(8→9)*abeo*-lanostane	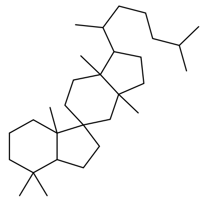	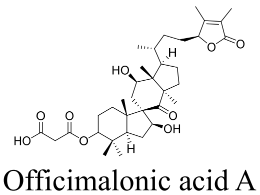 Officimalonic acid A	*Fomes officinalis*<br>*Phytochemistry*, **2016**, 130, 193-200<br>10.1016/j.phytochem.2016.05.004
21,24-*cyclo*-lanostane	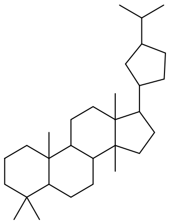	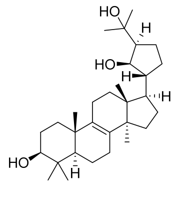 Inonotsutriol A	*Inonotus obliquus*<br>*Helv. Chim. Acta*, **2008**, 91, 1513-1524<br>10.1002/hlca.200890165

Tricholoma	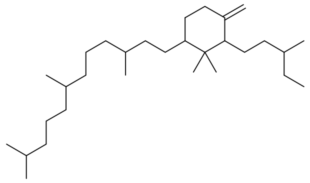	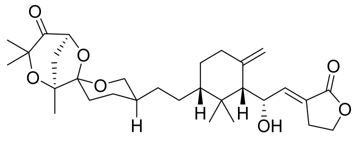 Terreolide A	*Tricholoma terreum* *Chem. Eur. J.*, **2014**, 20, 7001-7009 10.1002/chem.201400226

[a]If the scaffolds have not been designated a name in original publications, the general names are given here.

2. Supplementary figures.

Figure S1. ^{1}H NMR spectrum of **1** (600 MHz, C_5D_5N).

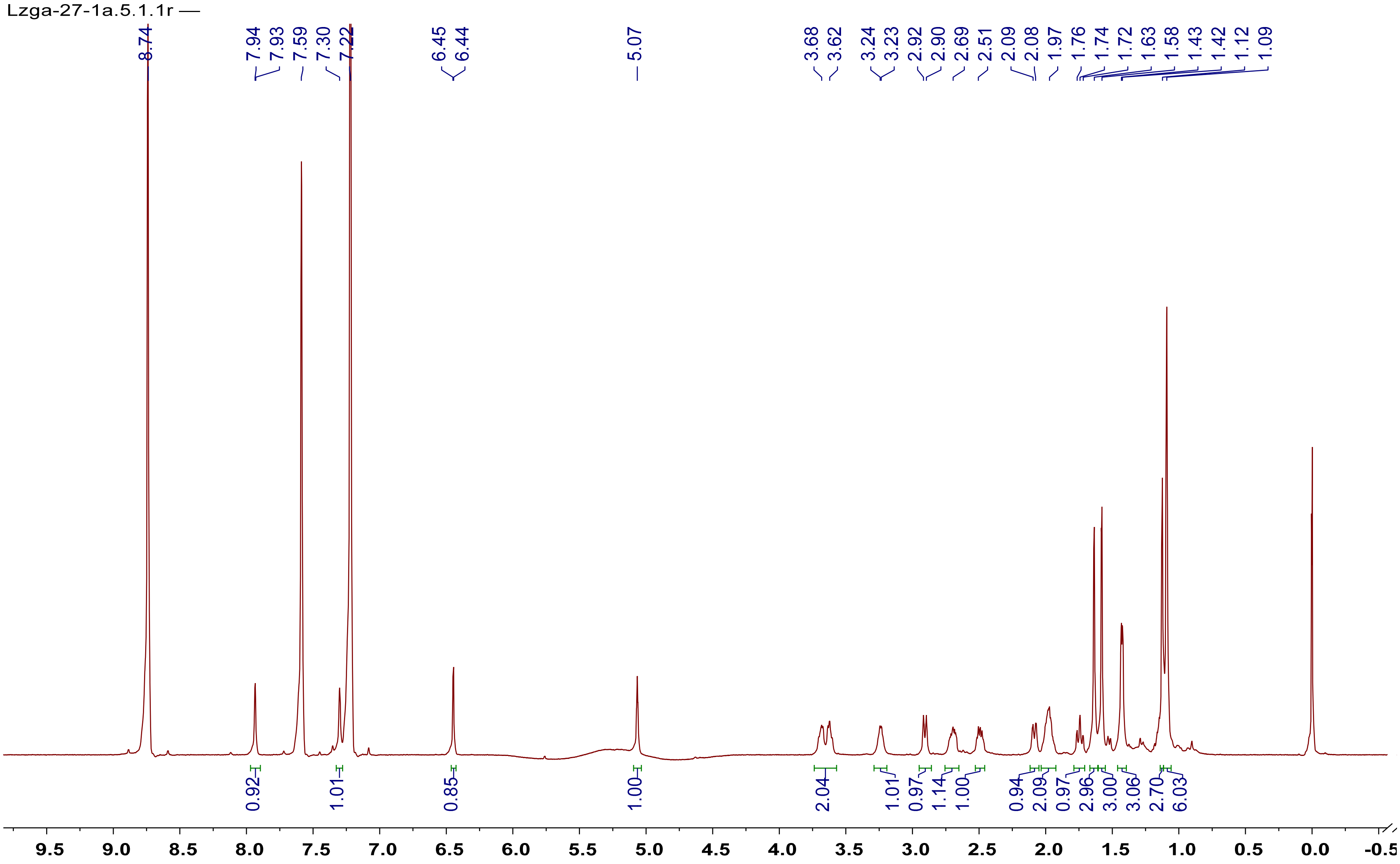

Figure S2. ^{13}C and DEPT NMR spectra of **1** (150 MHz, C_5D_5N).

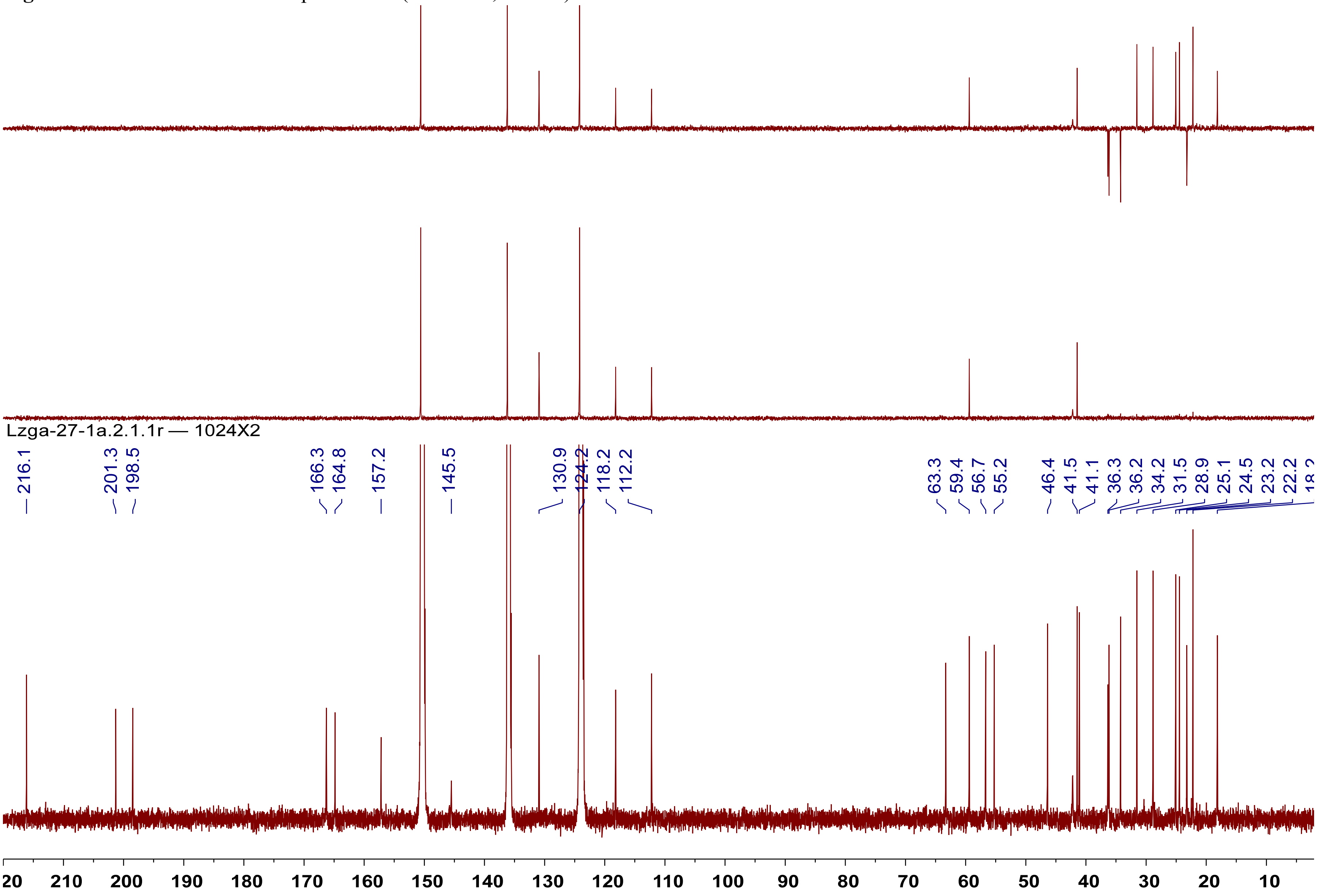

Figure S3. Enlarged ^{13}C and DEPT NMR spectra of **1** (150 MHz, C_5D_5N).

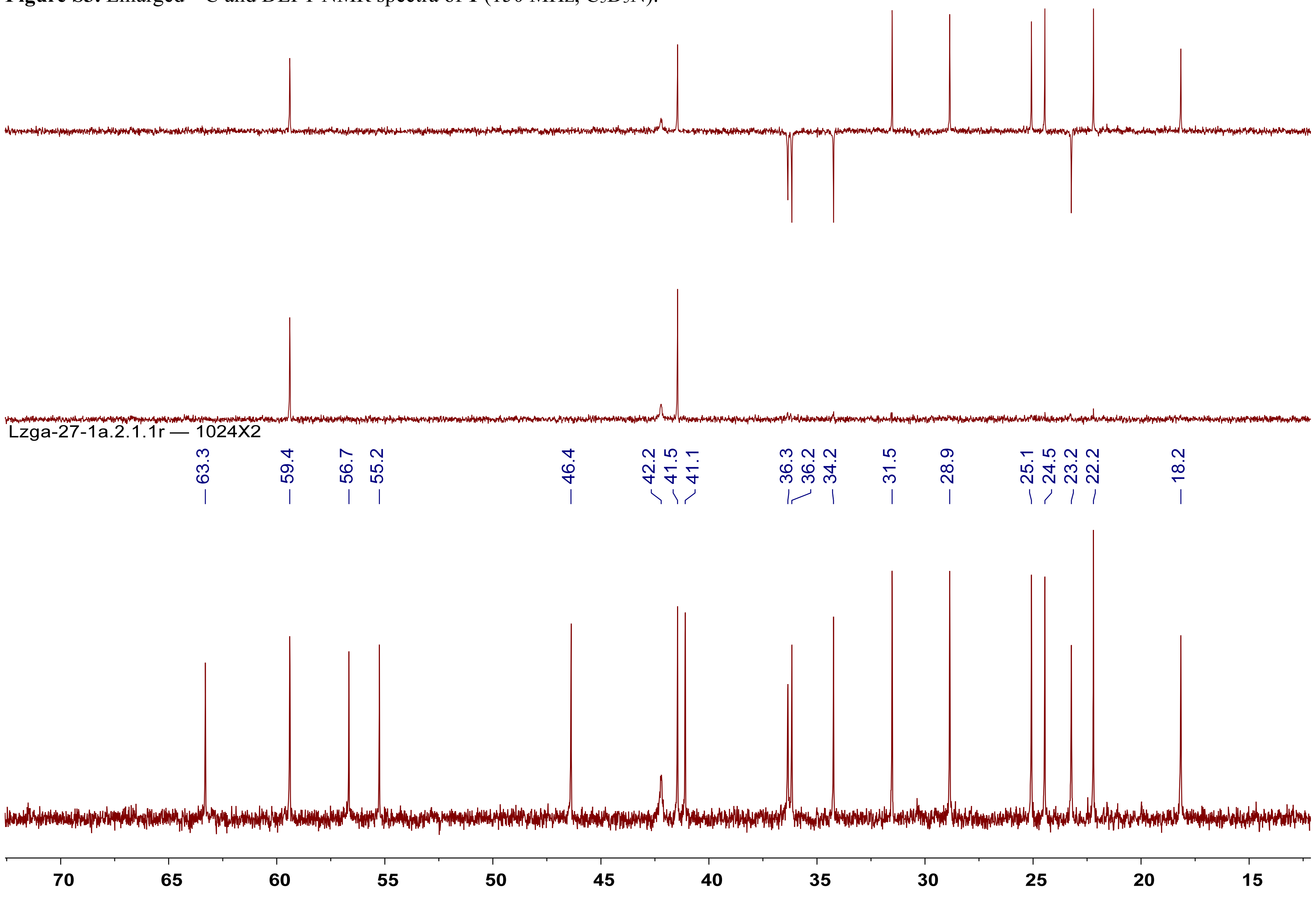

Figure S4. HSQC spectrum of **1** (C_5D_5N).

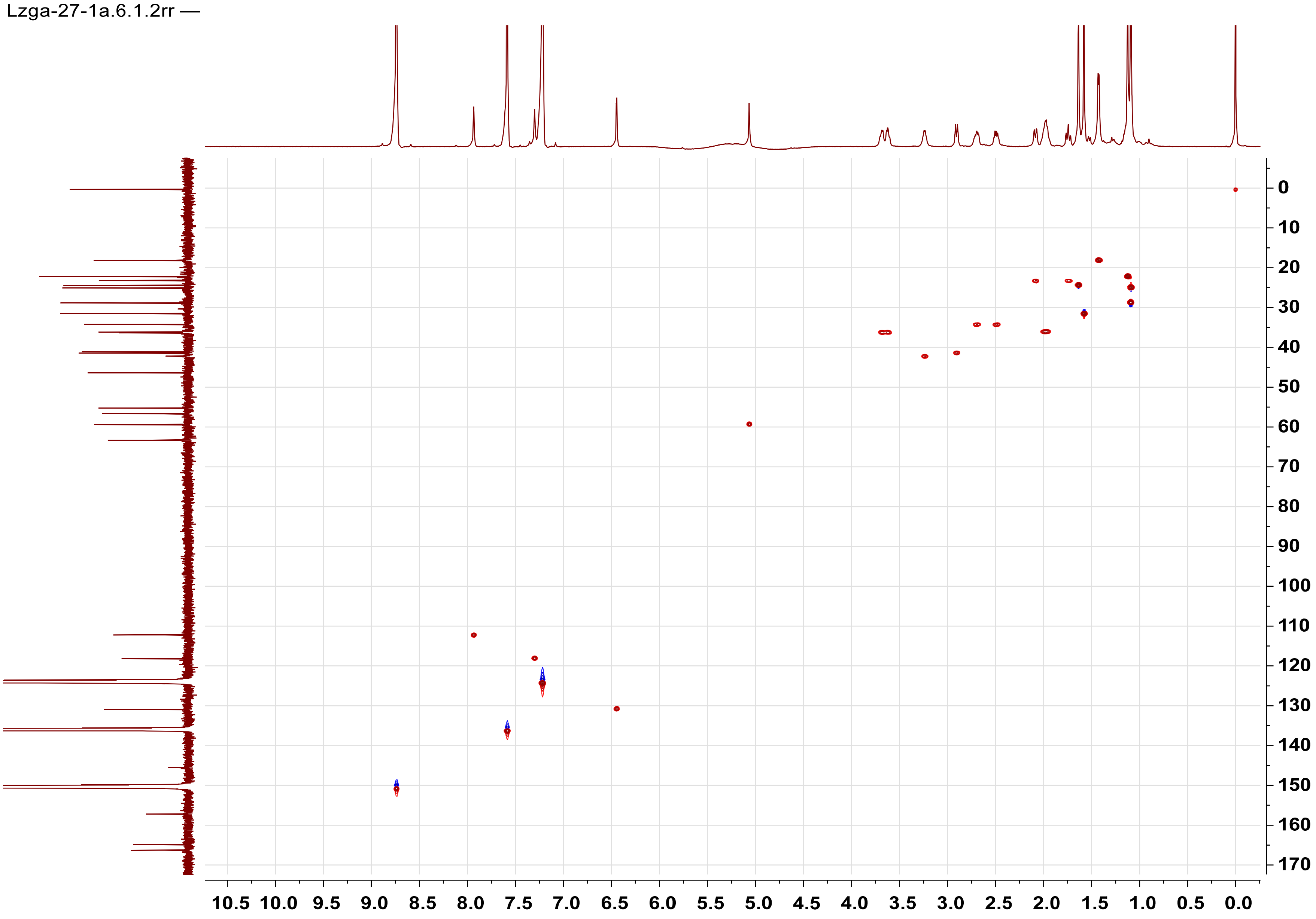

Figure S5. ^1H-^1H COSY spectrum of **1** (C_5D_5N).

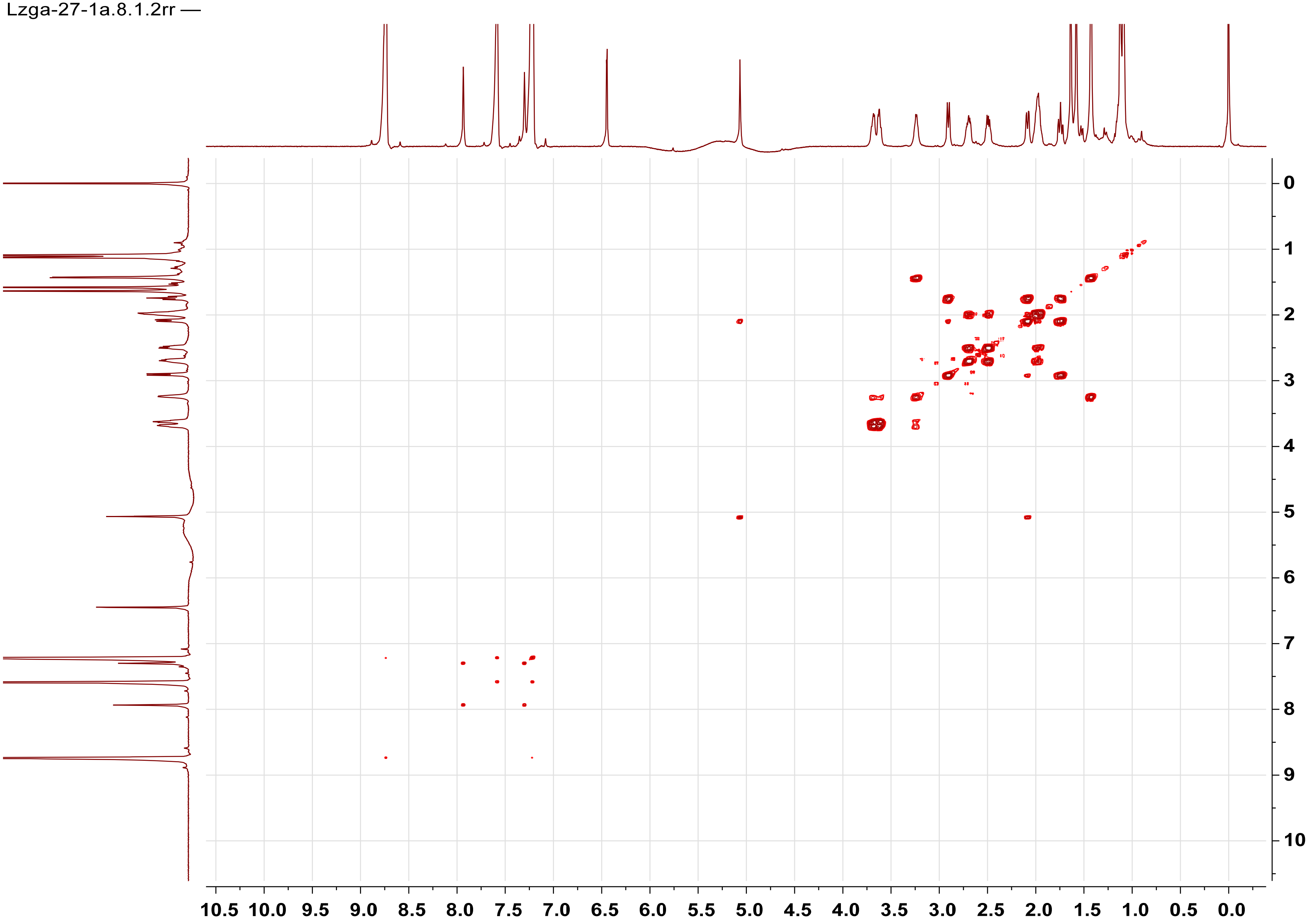

Figure S6. HMBC spectrum of **1** (C_5D_5N).

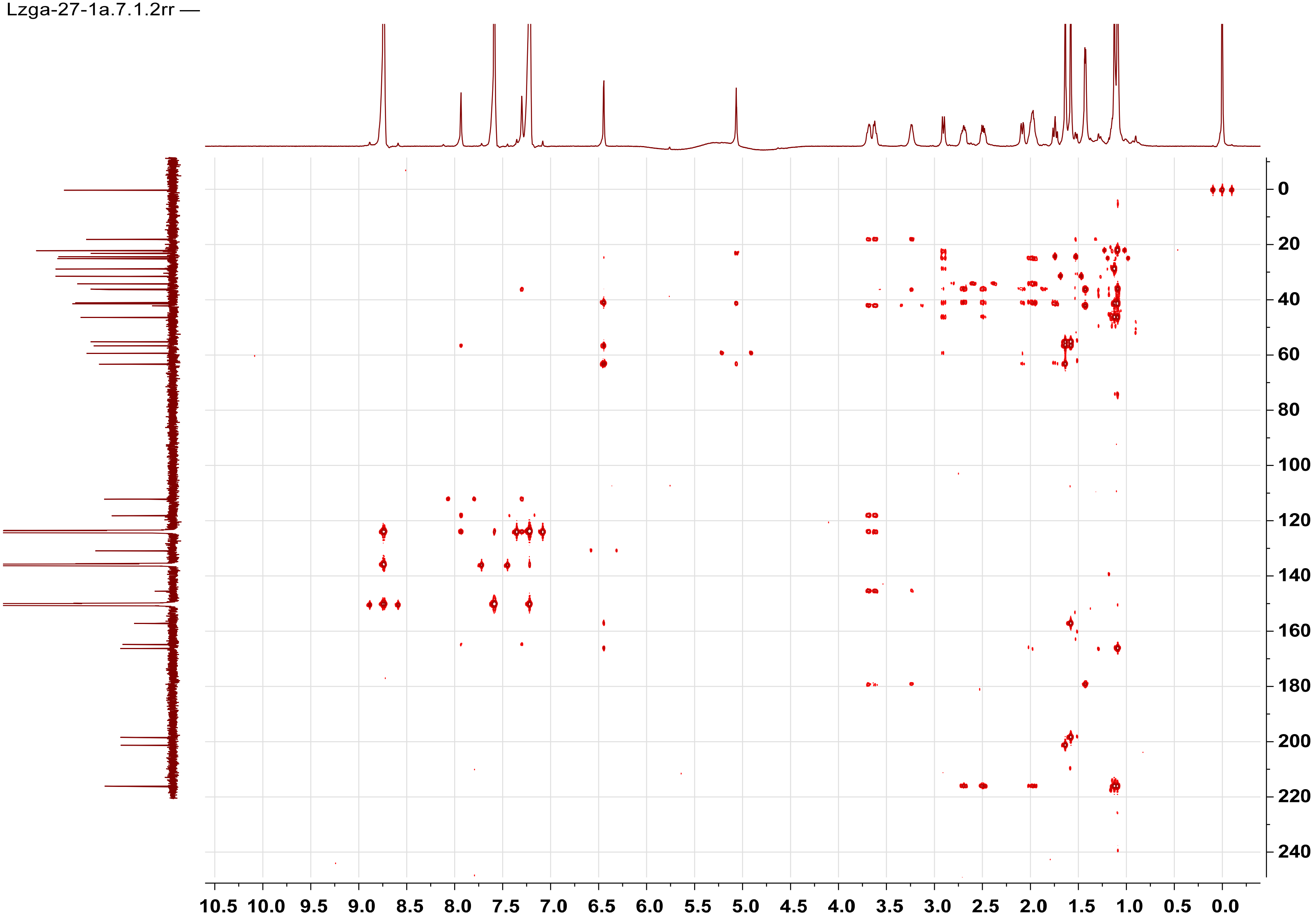

Figure S7. Enlarged HMBC spectrum A of **1** (C_5D_5N).

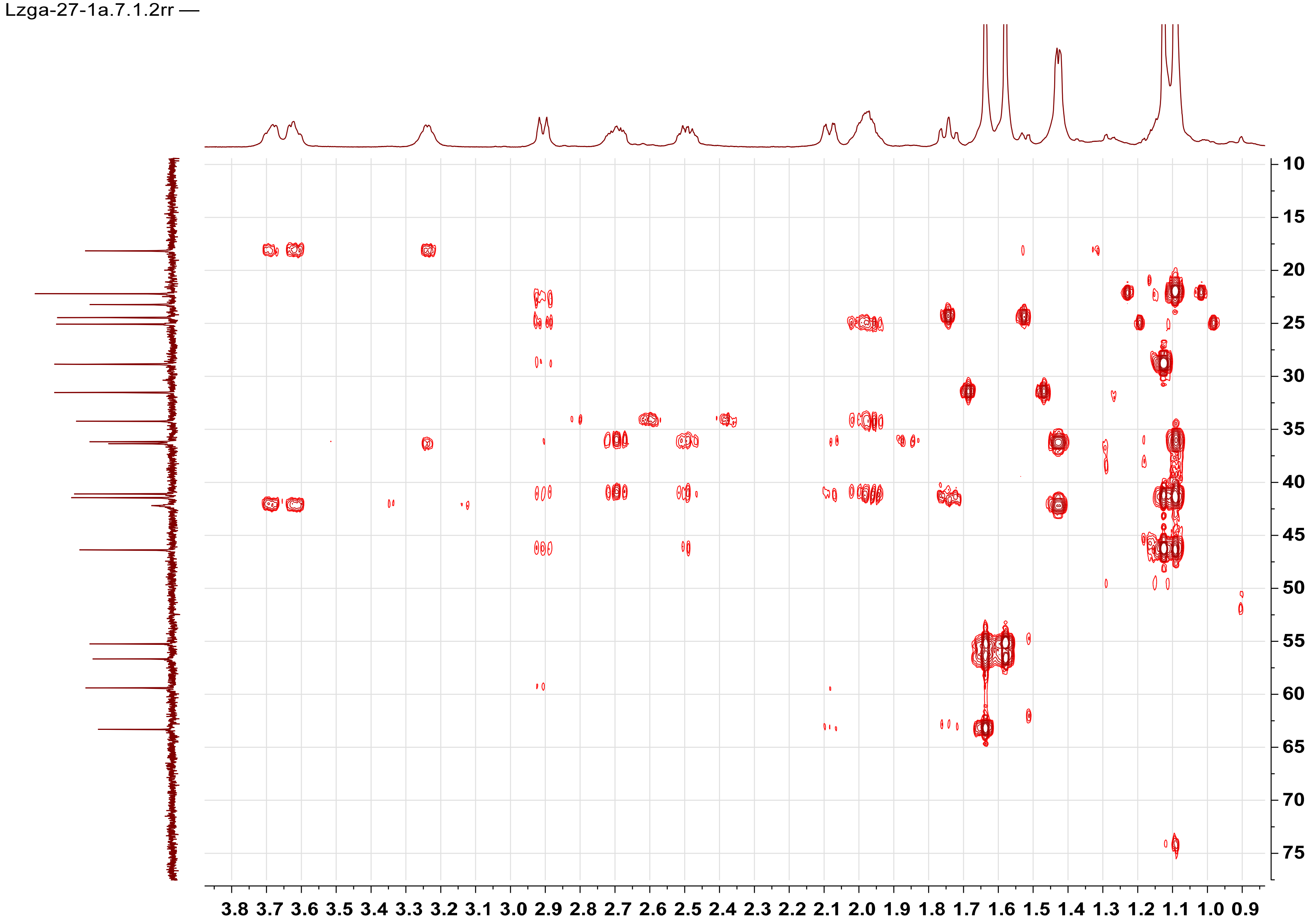

Figure S8. Enlarged HMBC spectrum B of **1** (C_5D_5N).

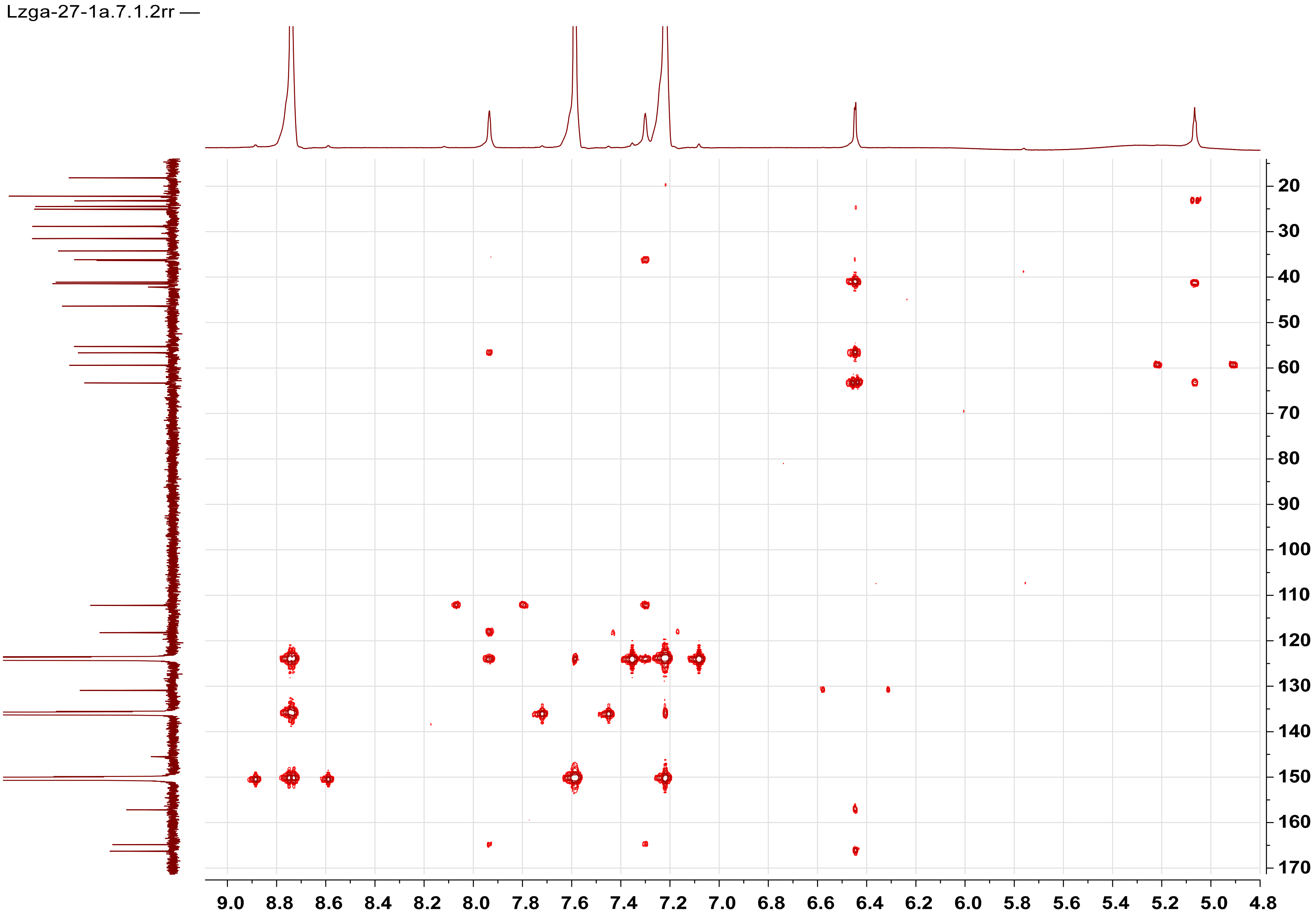

Figure S9. ROESY spectrum of **1** (C_5D_5N).

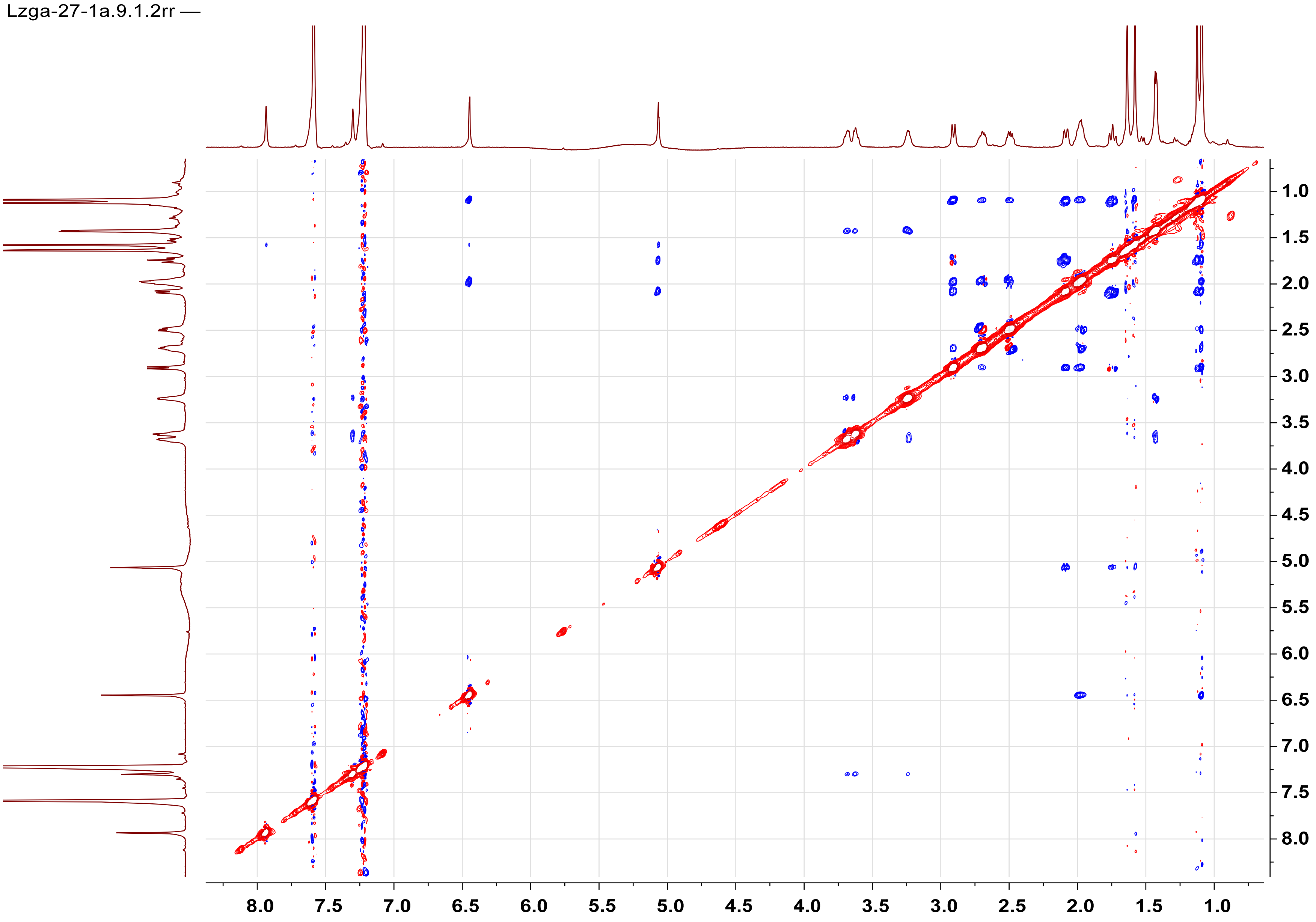

Figure S10. ^{1}H NMR spectrum of **1** (600 MHz, $CDCl_3$).

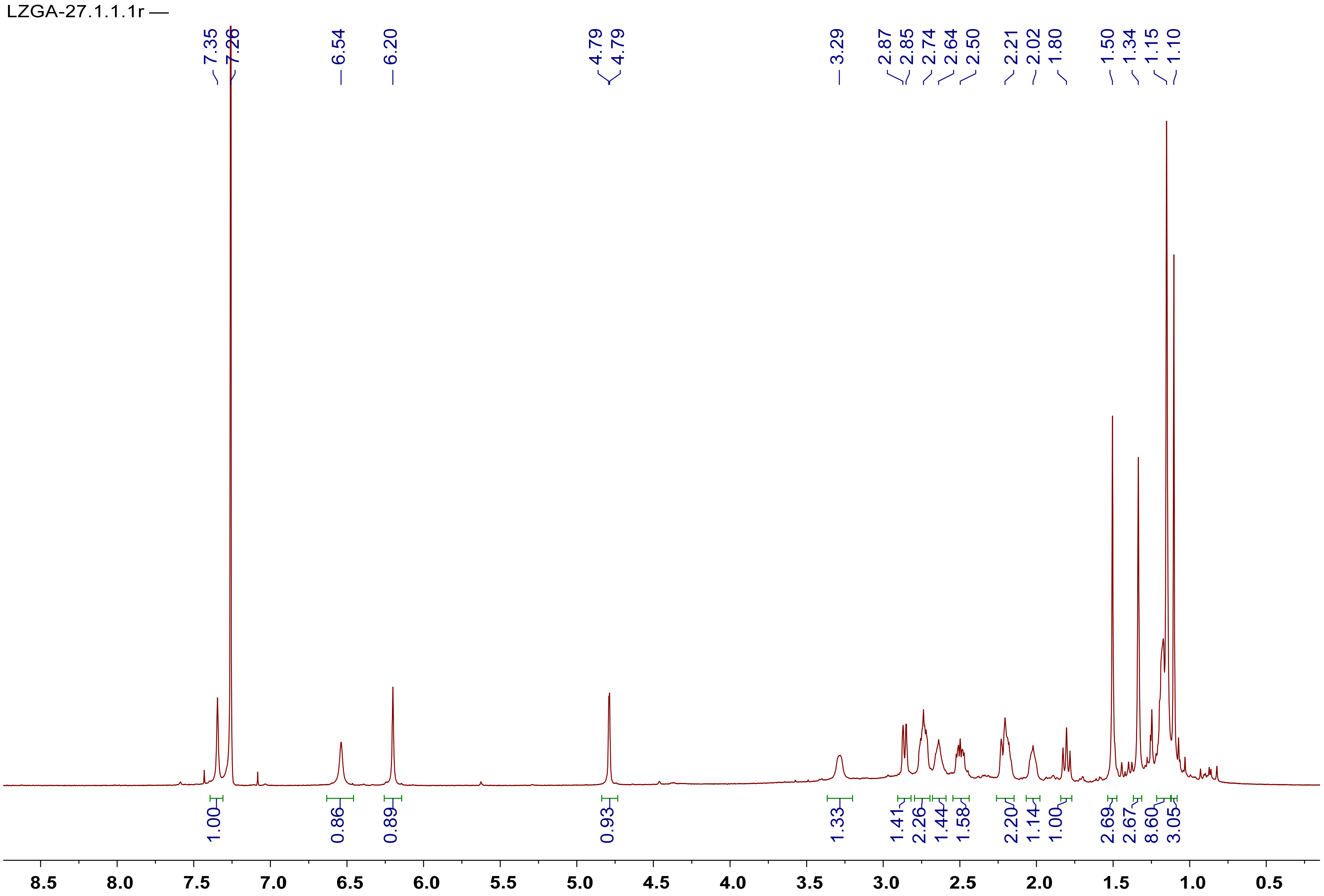

Figure S11. ^{13}C and DEPT NMR spectra of **1** (150 MHz, $CDCl_3$).

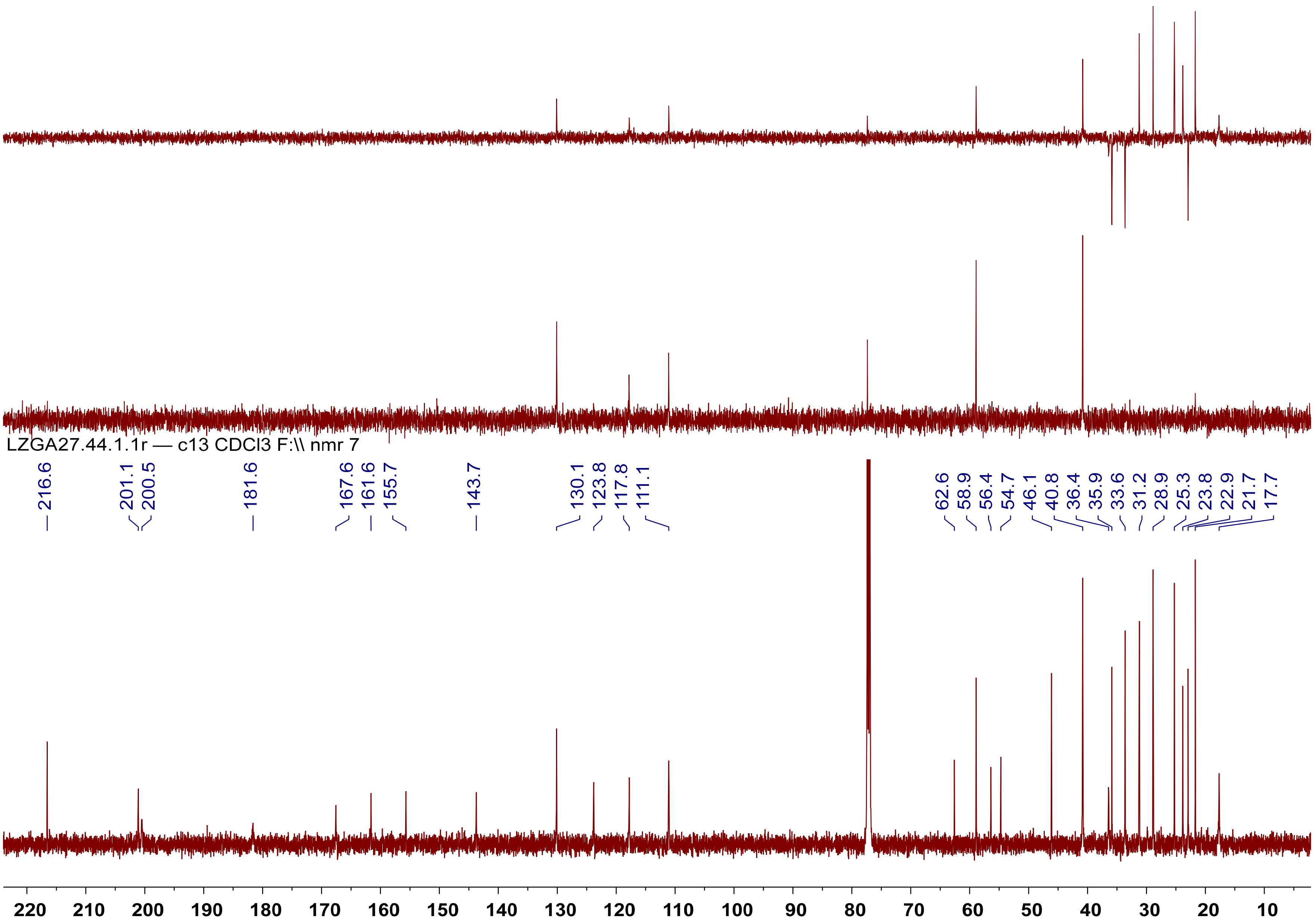

Figure S12. Enlarged ^{13}C and DEPT NMR spectra of **1** (150 MHz, $CDCl_3$).

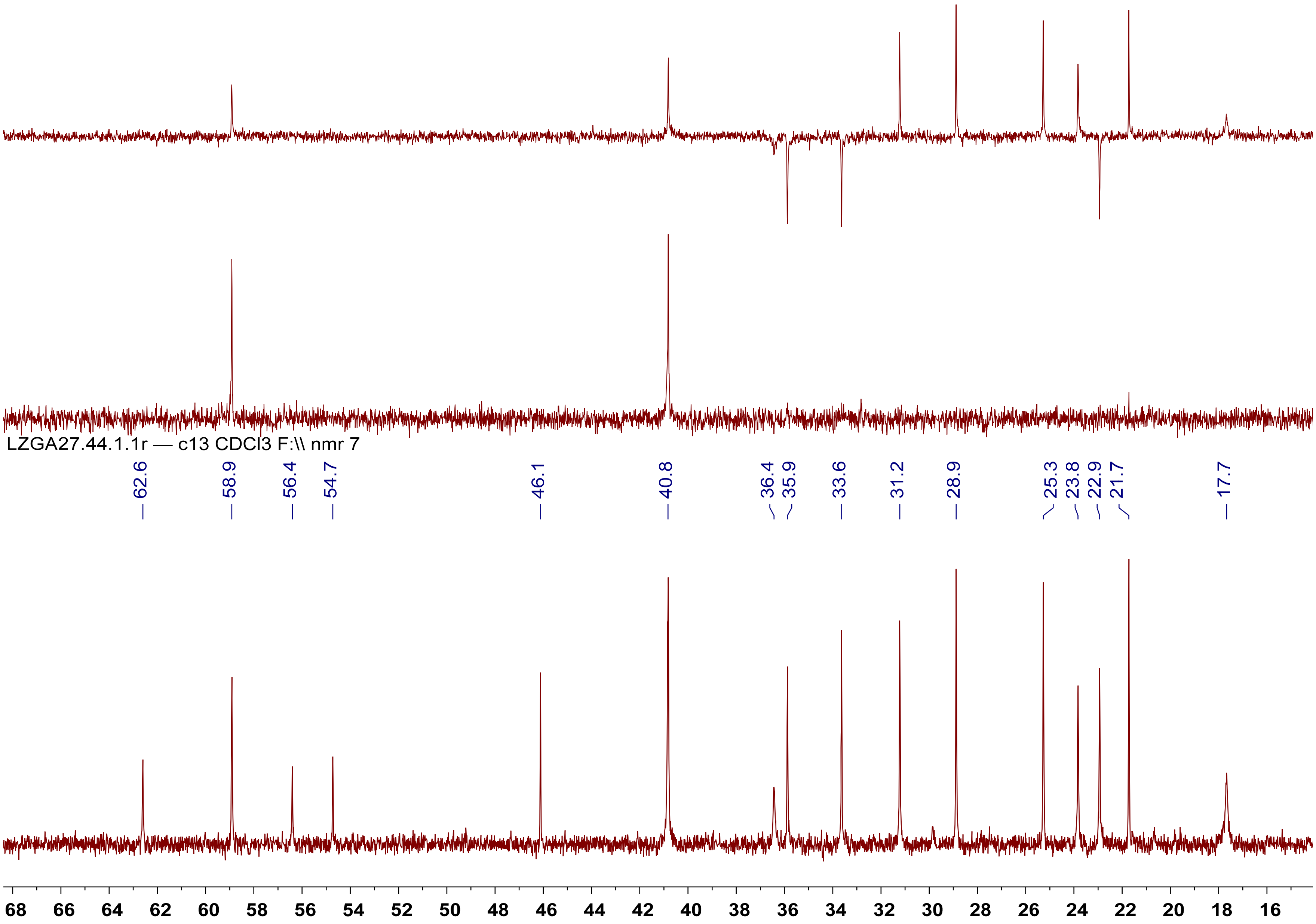

Figure S13. HSQC spectrum of **1** ($CDCl_3$).

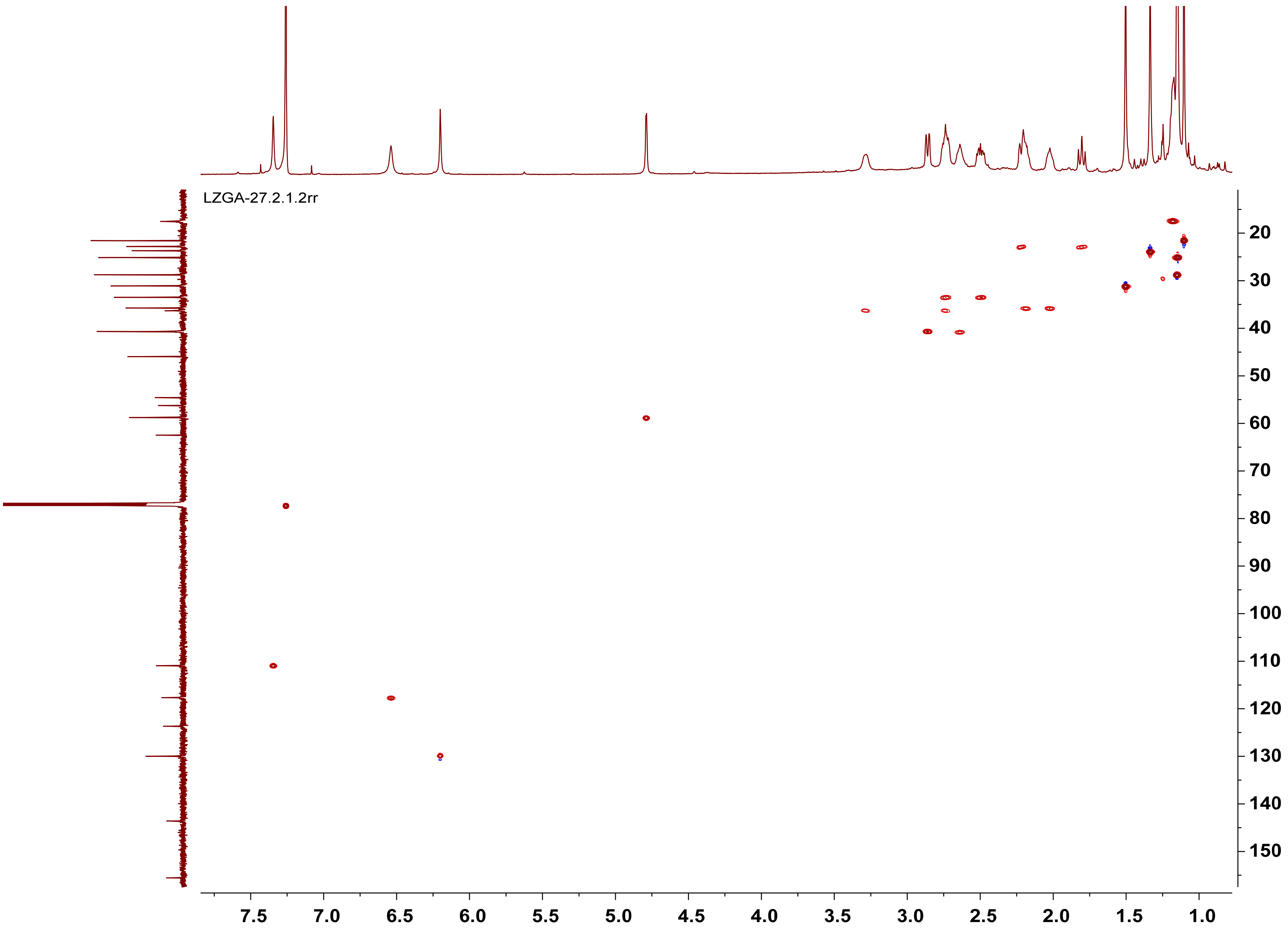

Figure S14. ^{1}H-^{1}H COSY spectrum of **1** ($CDCl_3$).

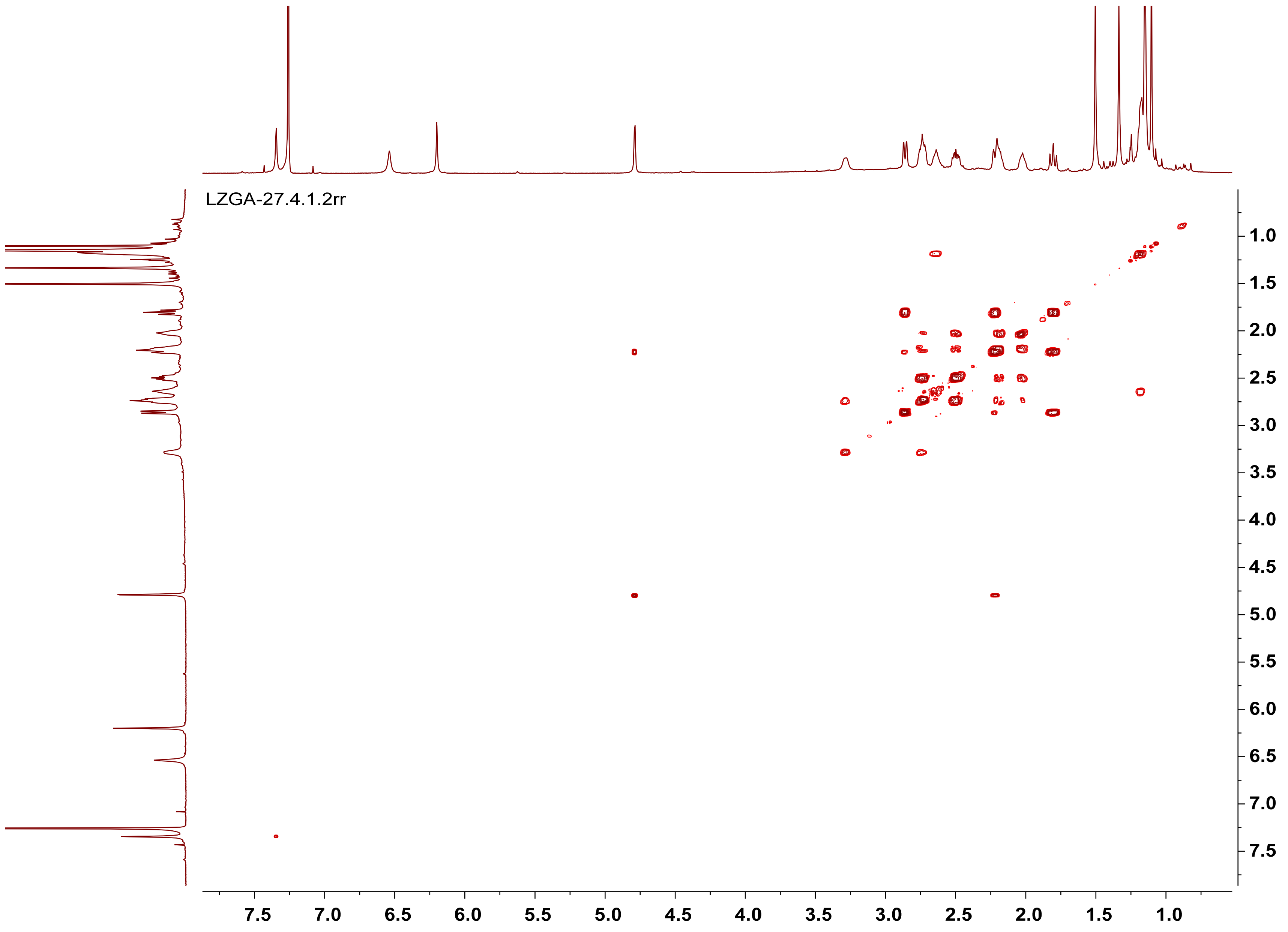

Figure S15. HMBC spectrum of **1** ($CDCl_3$).

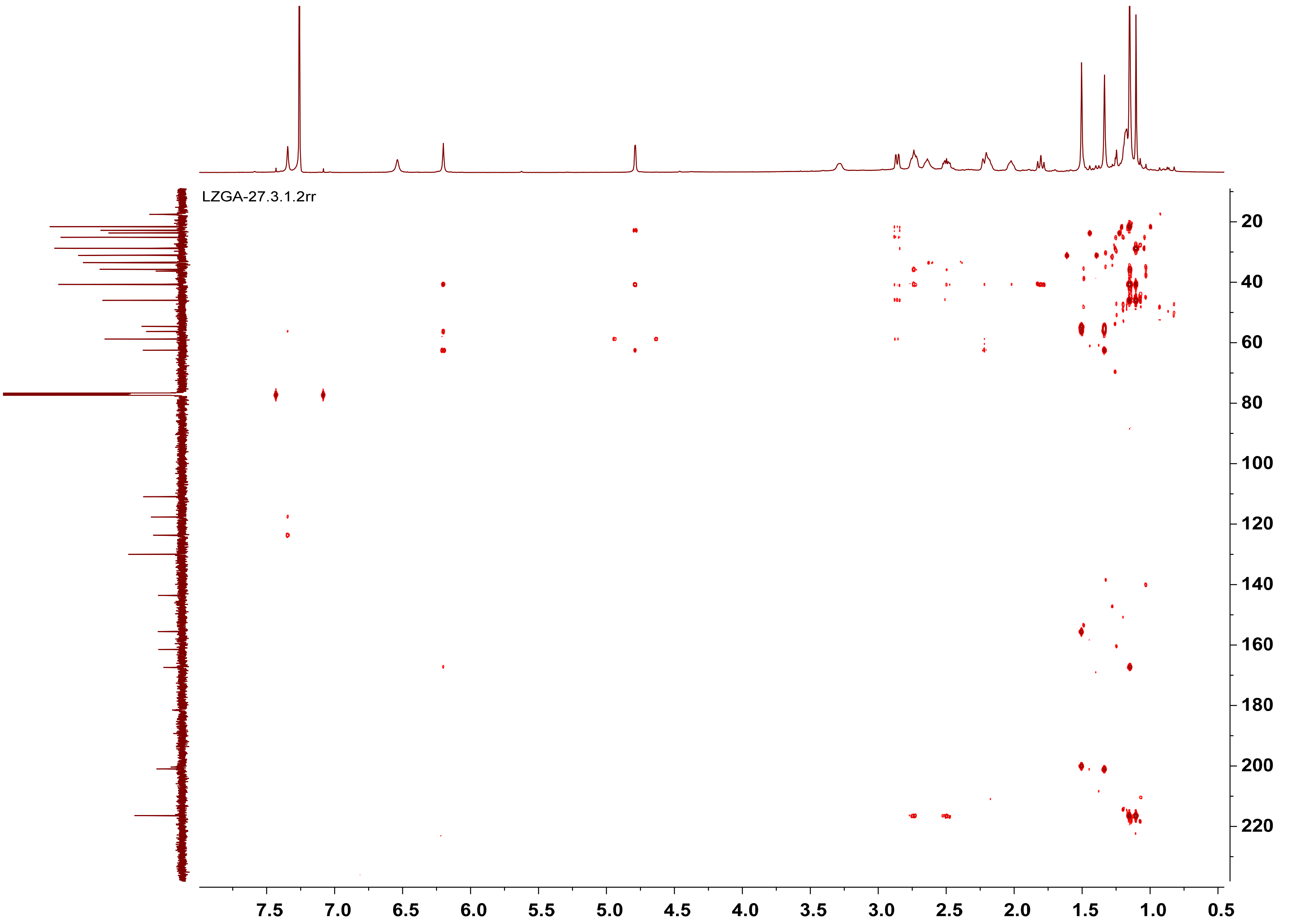

Figure S16. Enlarged HMBC spectrum A of **1** ($CDCl_3$).

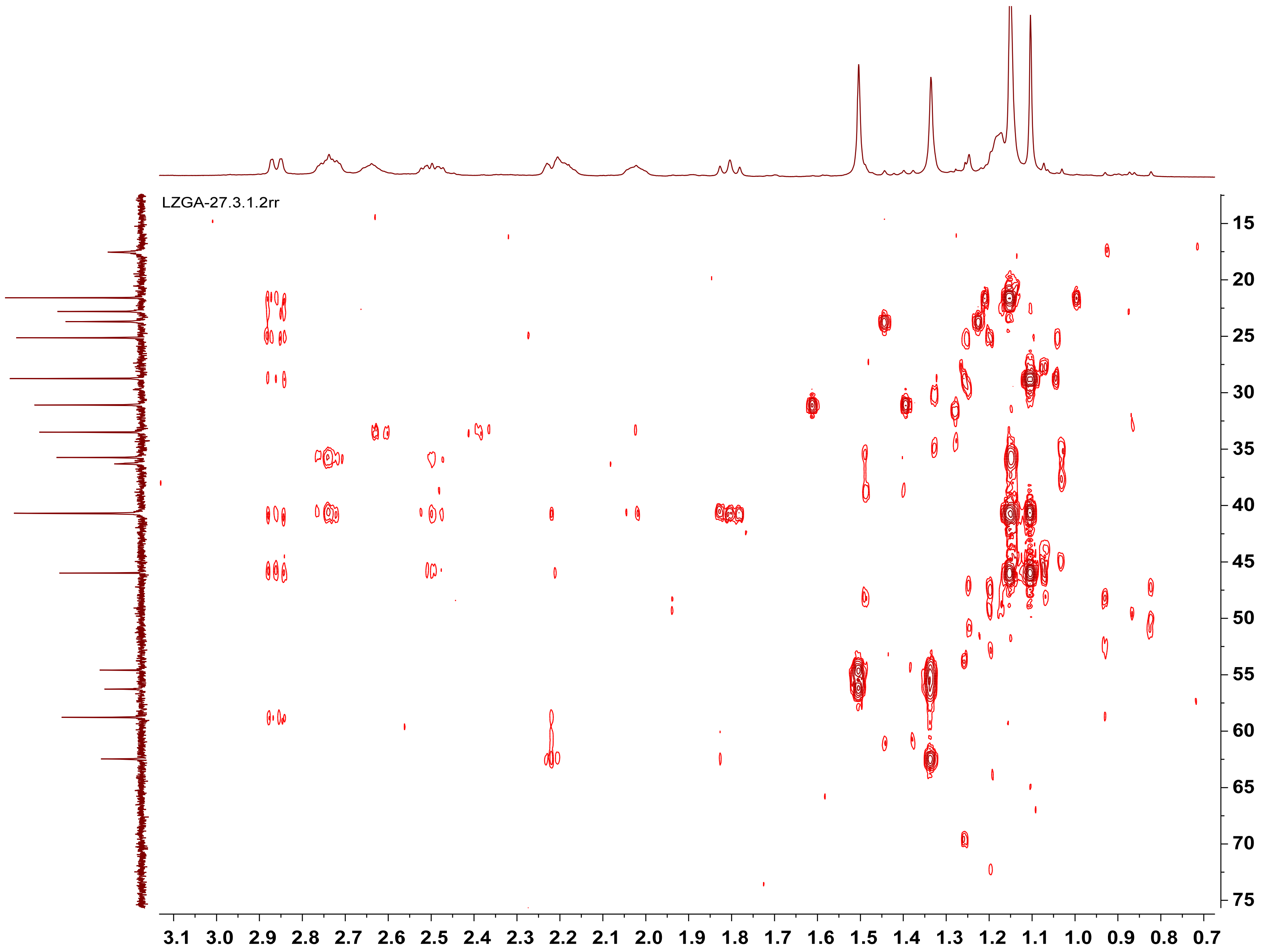

Figure S17. ROESY spectrum of **1** ($CDCl_3$).

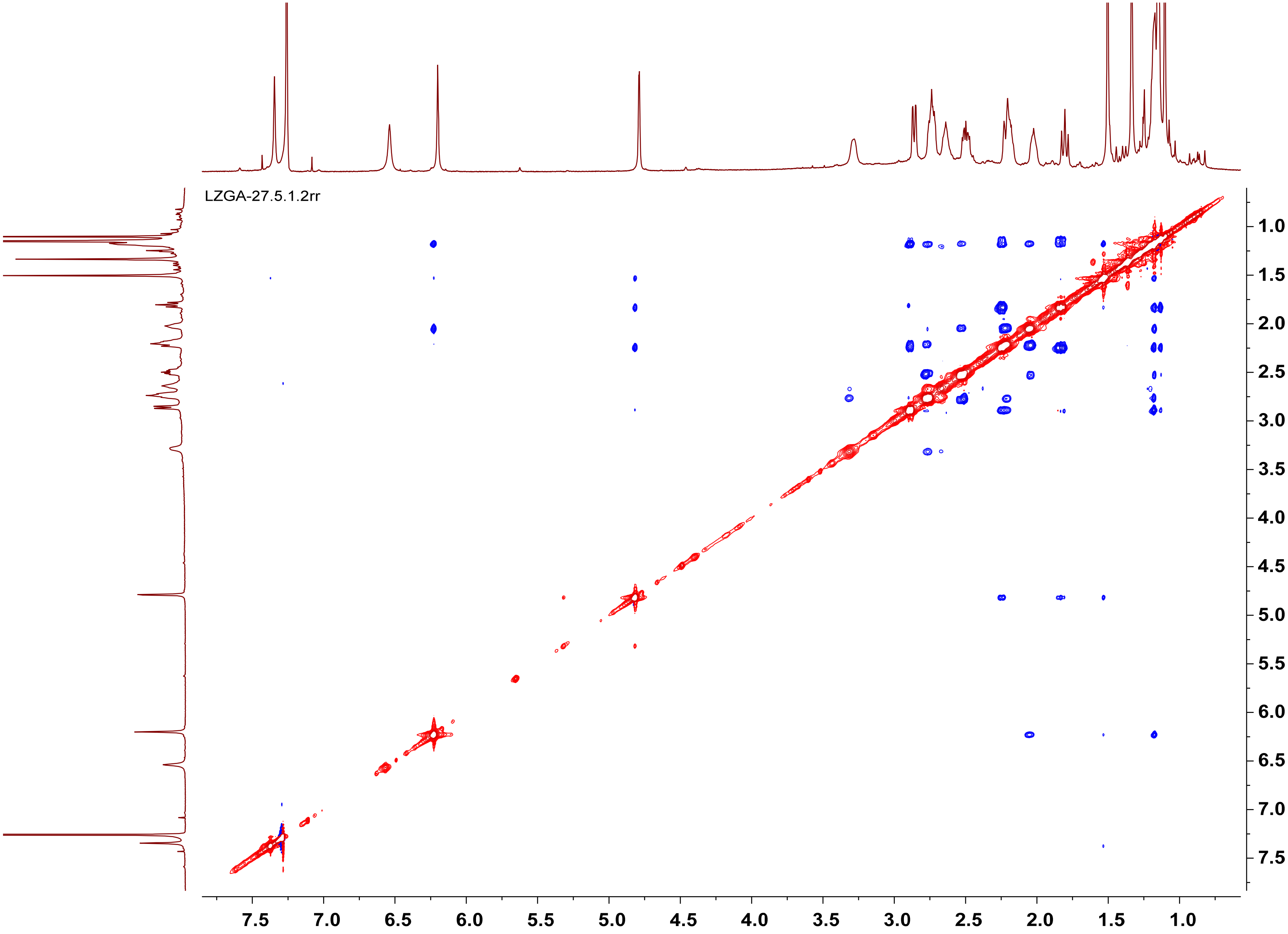

Figure S18. HRESIMS report of **1**.

D:\spectrum\...\lzga-27_200917094544   2020/9/17 9:47:02

lzga-27_200917094544 #11 RT: 0.14 AV: 1 NL: 8.64E7
T: FTMS + p ESI Full lock ms [150.0000-2000.0000]

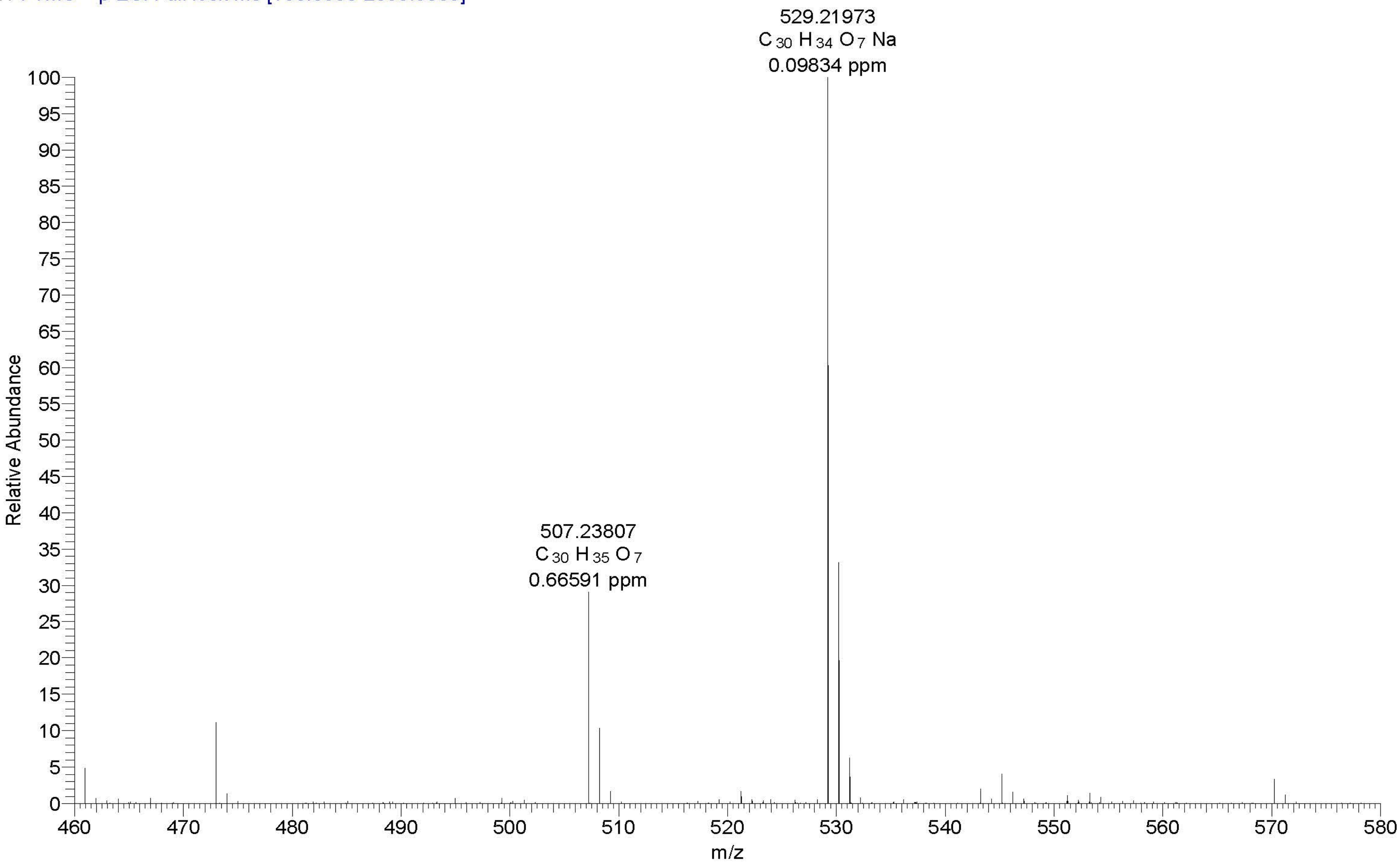

Figure S19. ^{1}H NMR spectrum of **2** (600 MHz, $CDCl_3$).

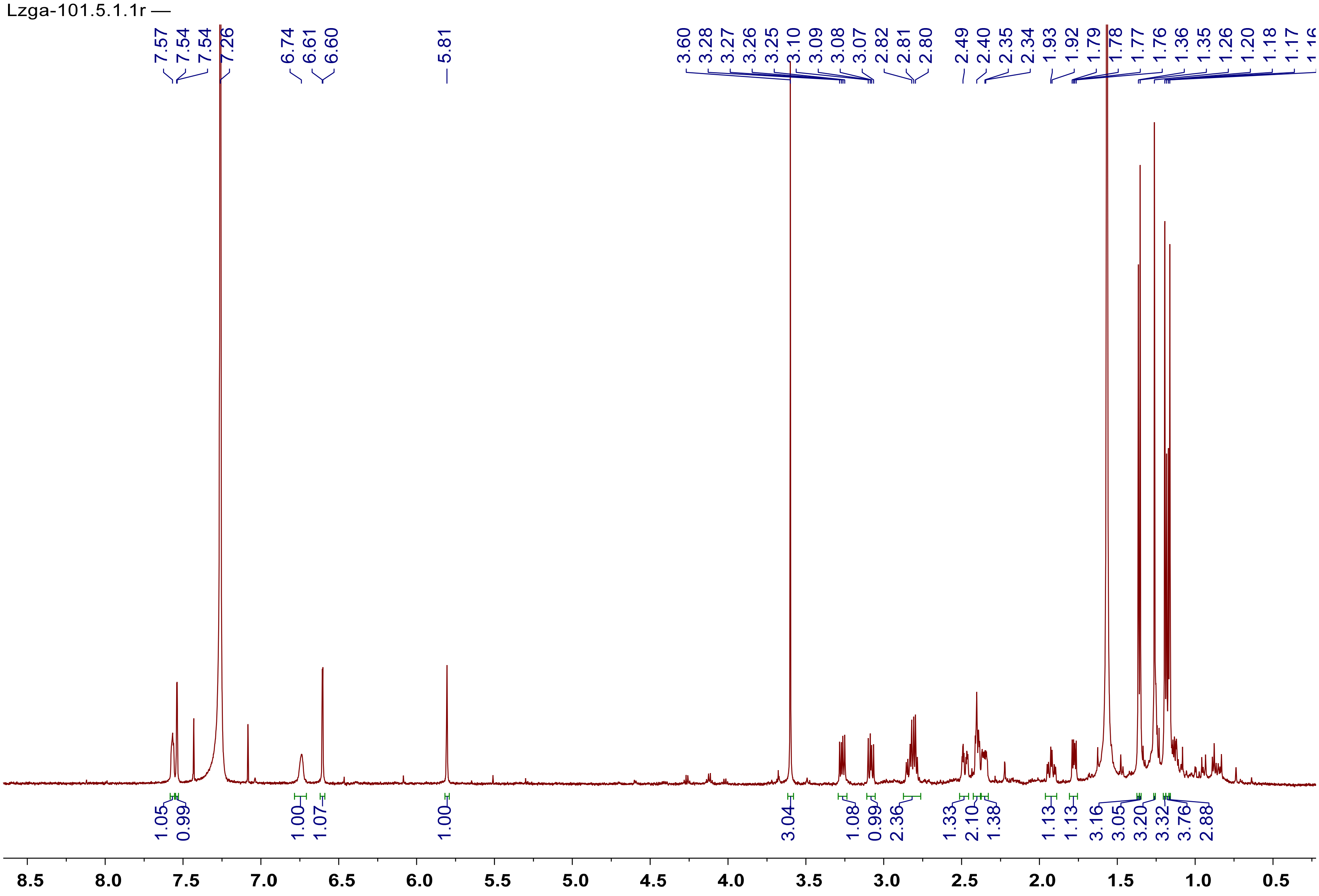

Figure S20. ^{13}C and DEPT135 NMR spectra of **2** (150 MHz, $CDCl_3$).

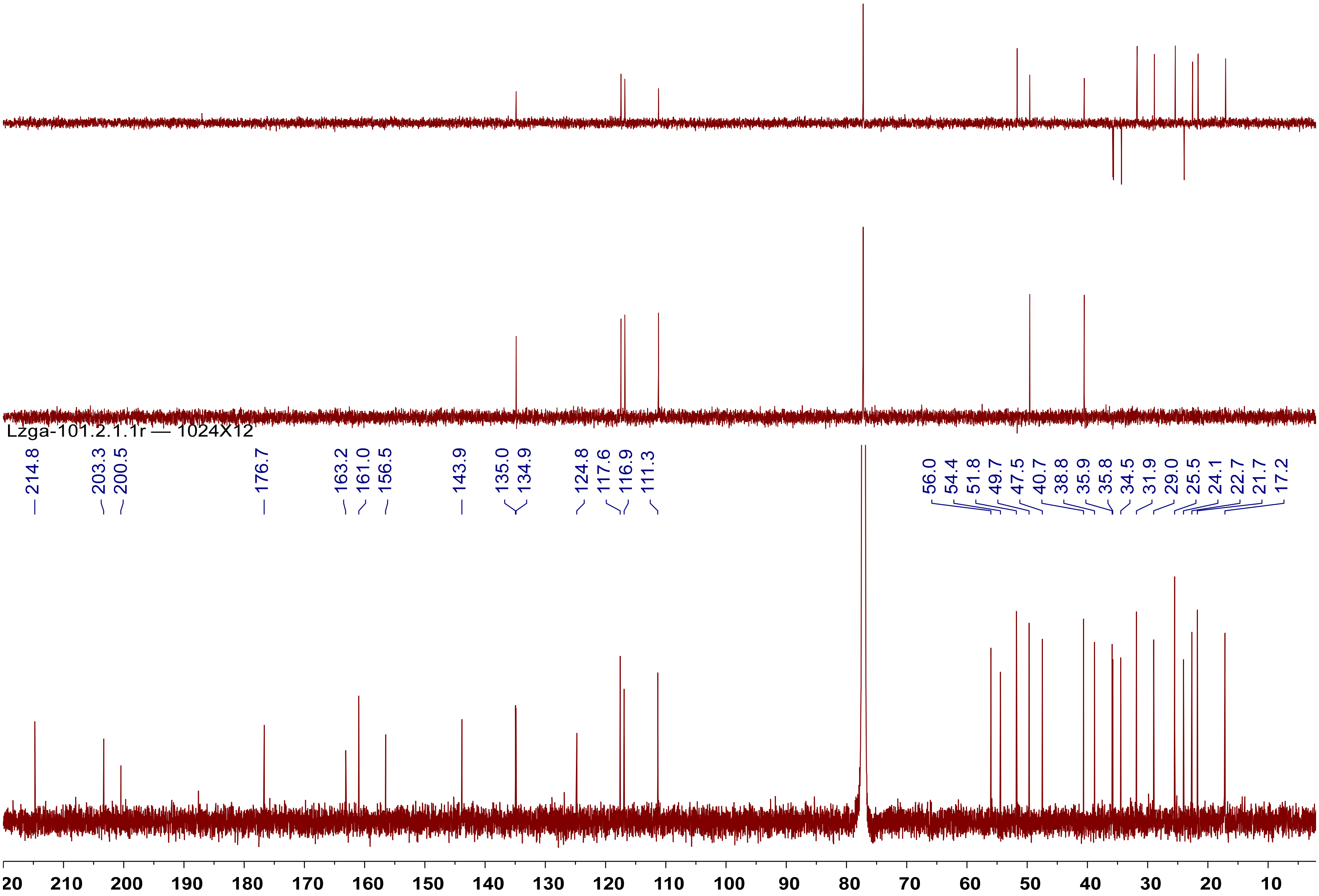

Figure S21. Enlarged ^{13}C and DEPT135 NMR spectra of **2** (150 MHz, $CDCl_3$).

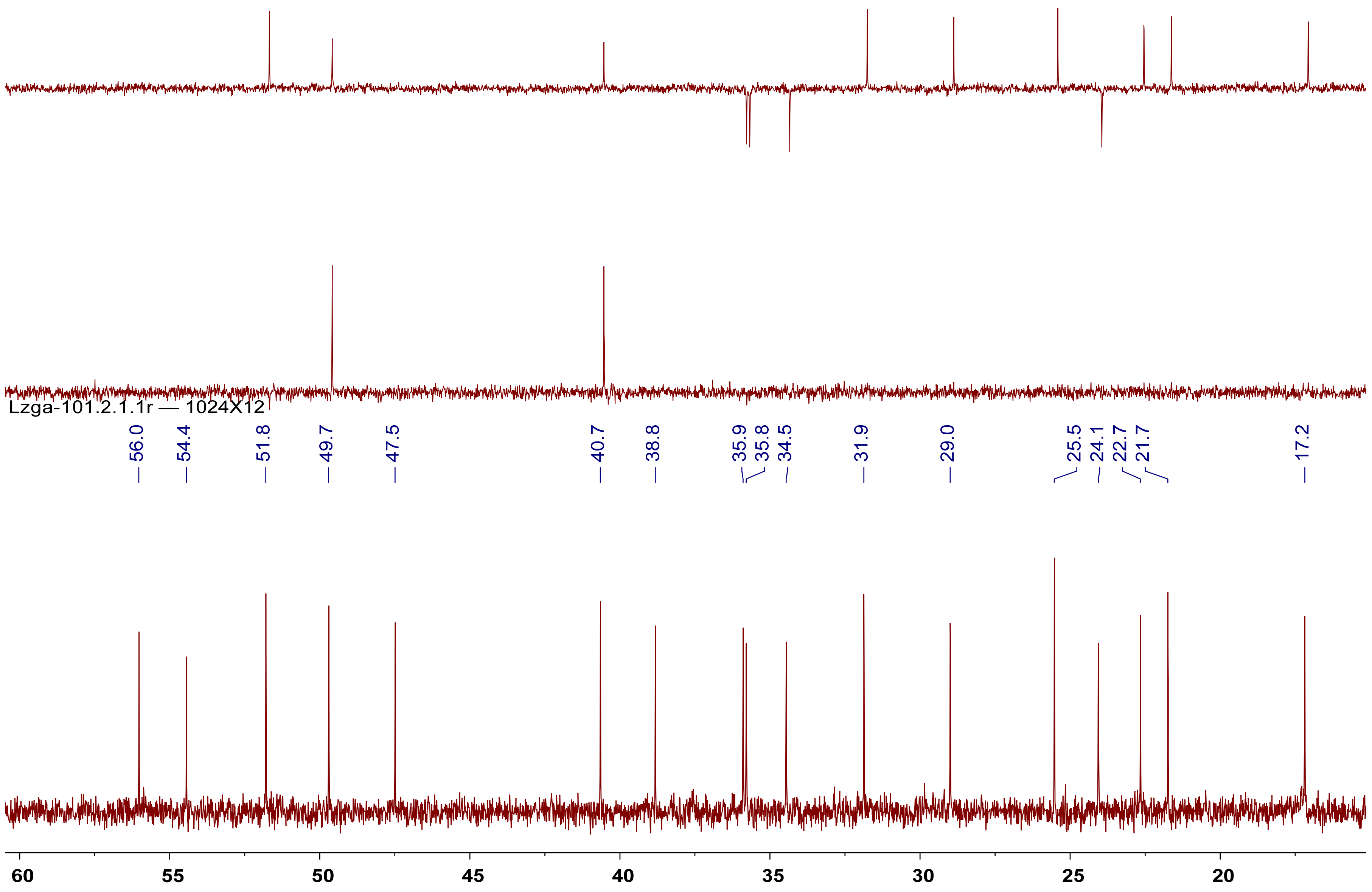

Figure S22. HSQC spectrum of **2**.

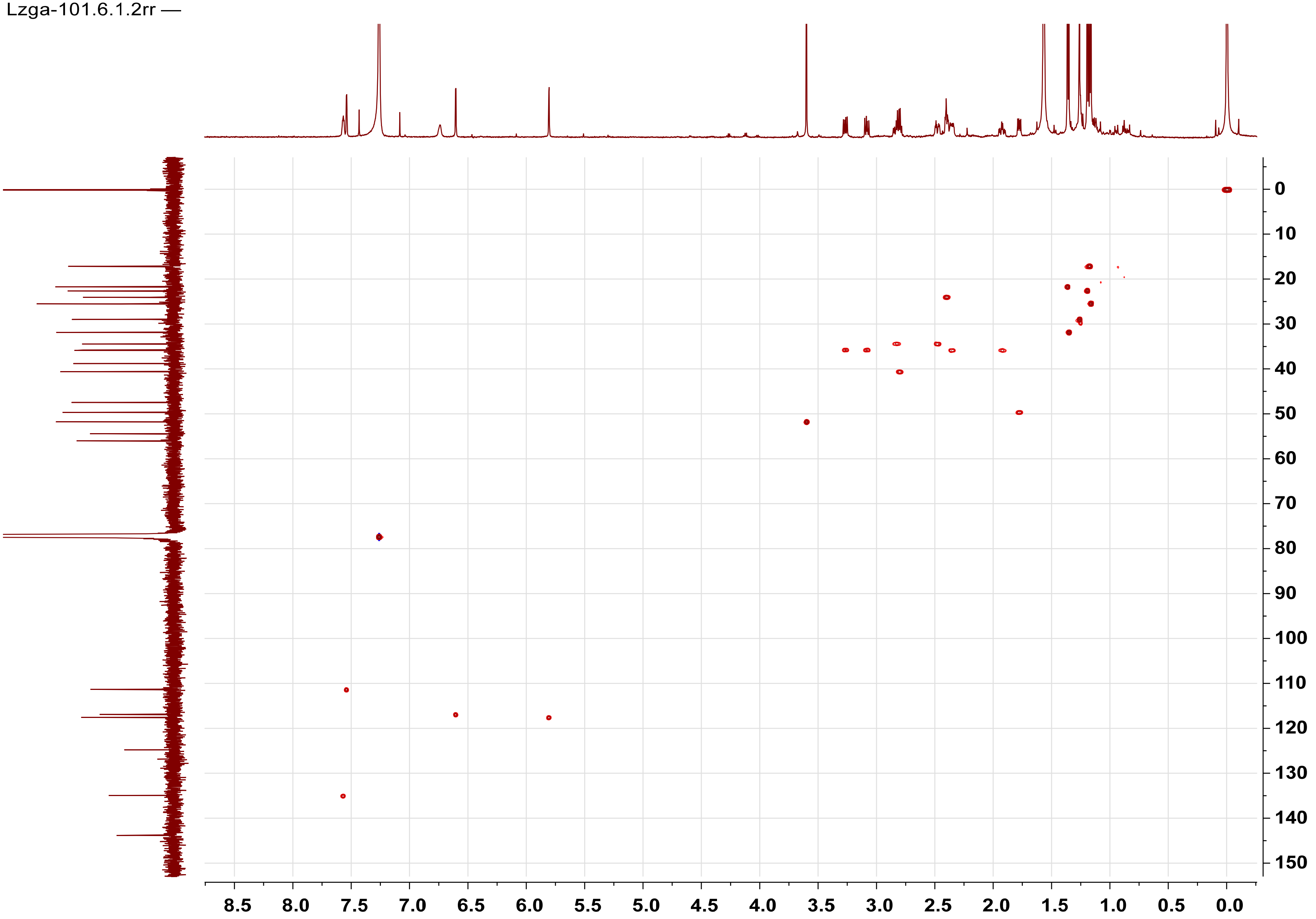

Figure S23. ^{1}H-^{1}H COSY spectrum of **2**.

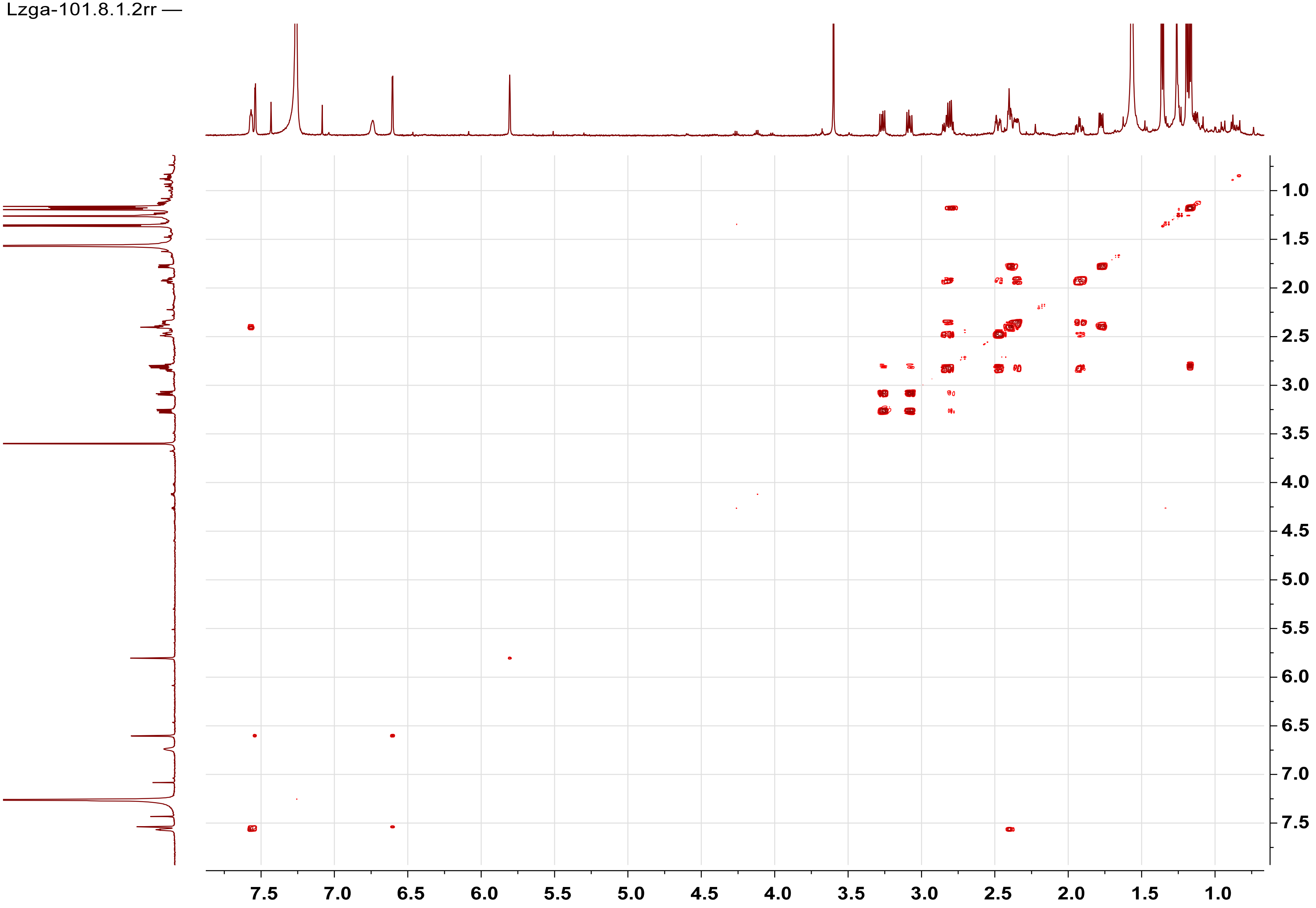

Figure S24. HMBC spectrum of **2**.

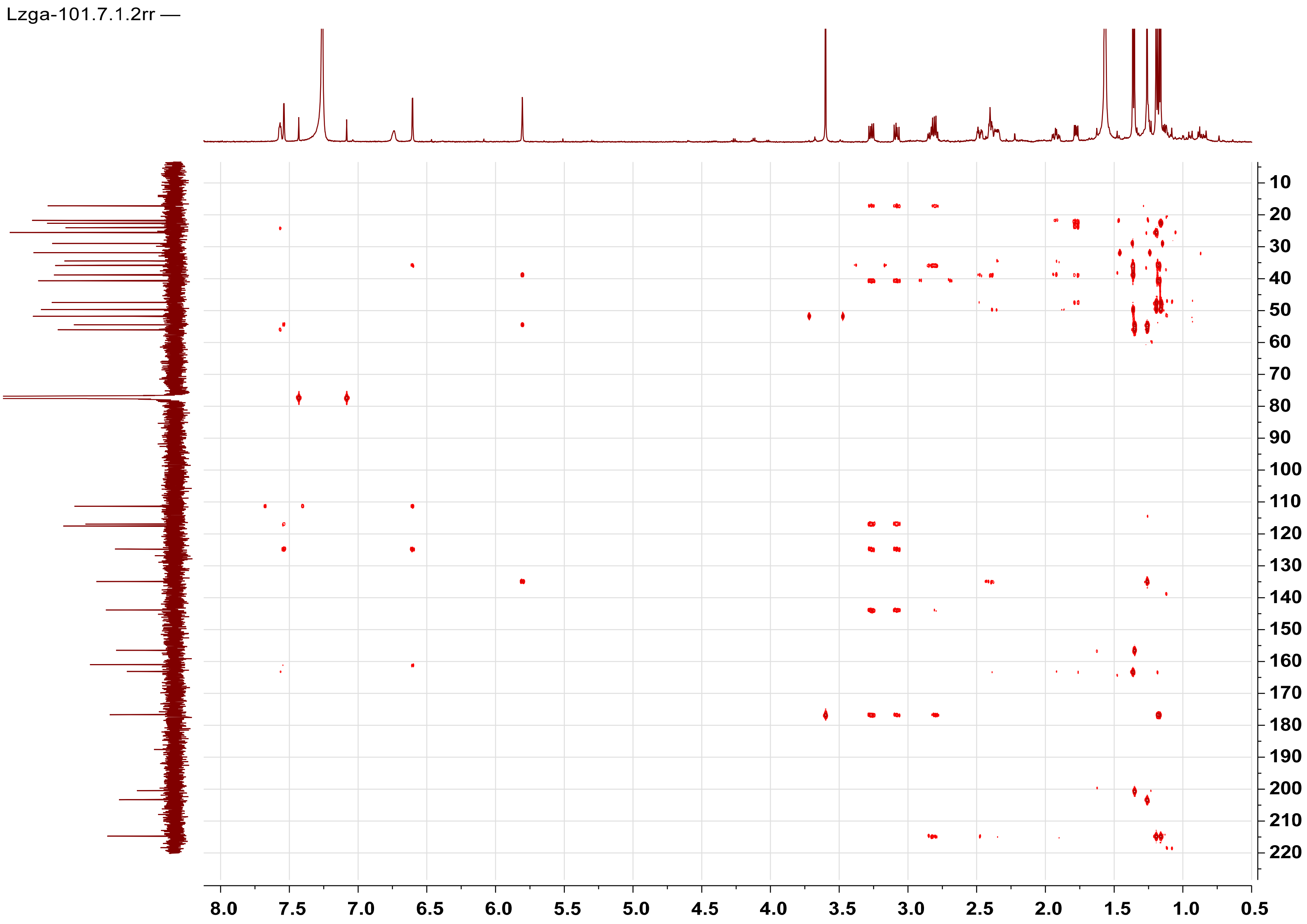

Figure S25. Enlarged HMBC spectrum A of **2**.

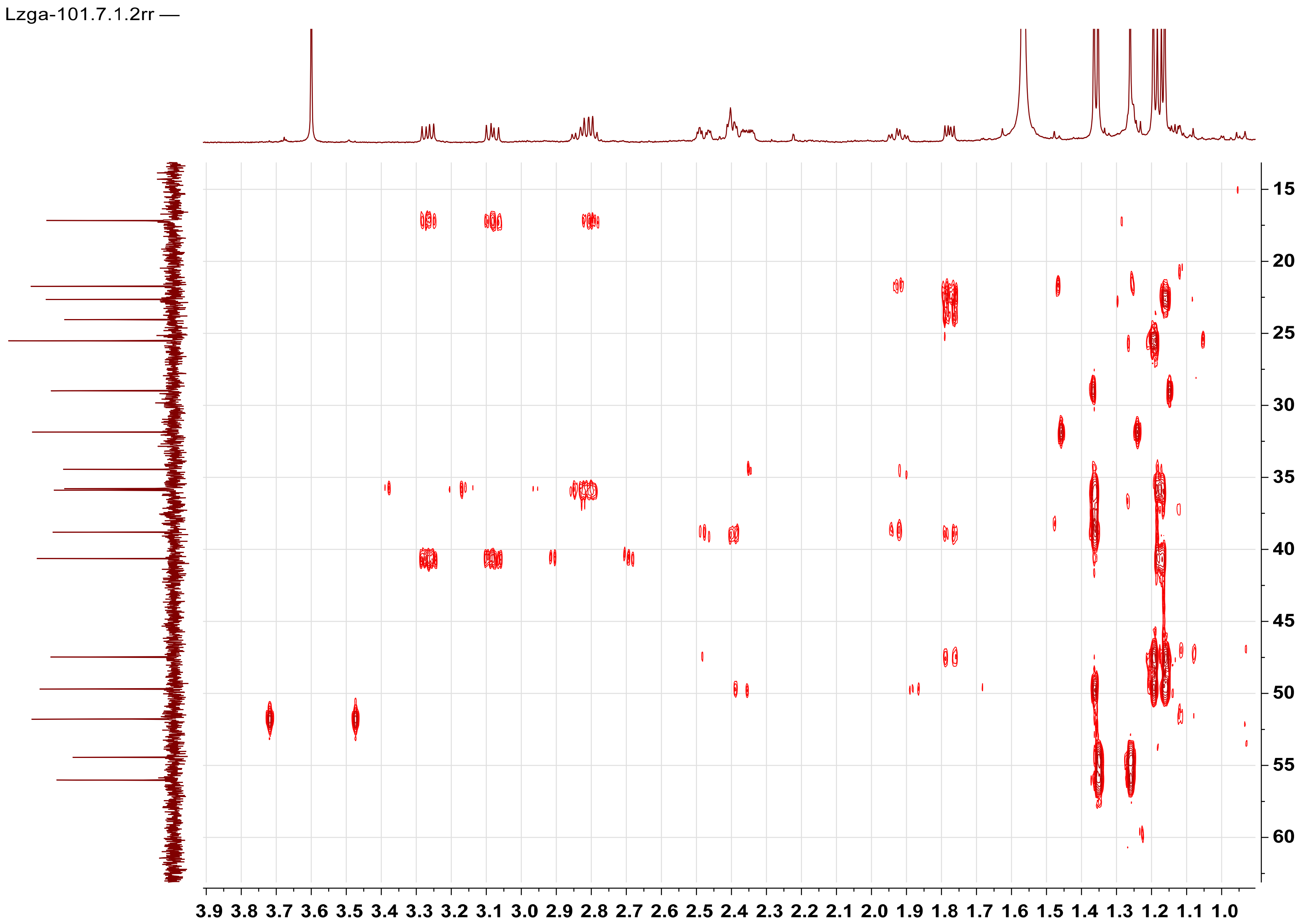

Figure S26. Enlarged HMBC spectrum B of **2**.

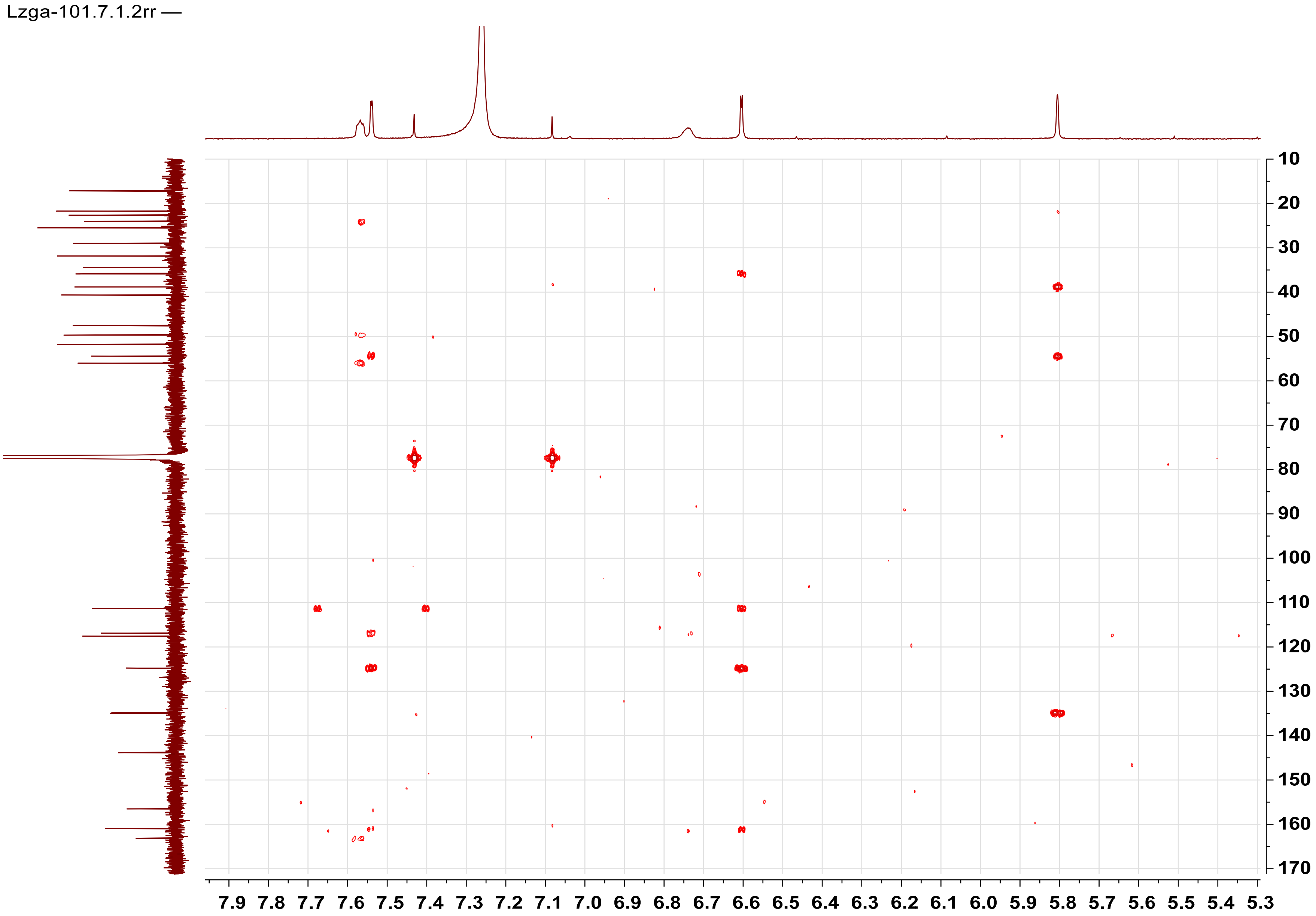

Figure S27. ROESY spectrum of **2**.

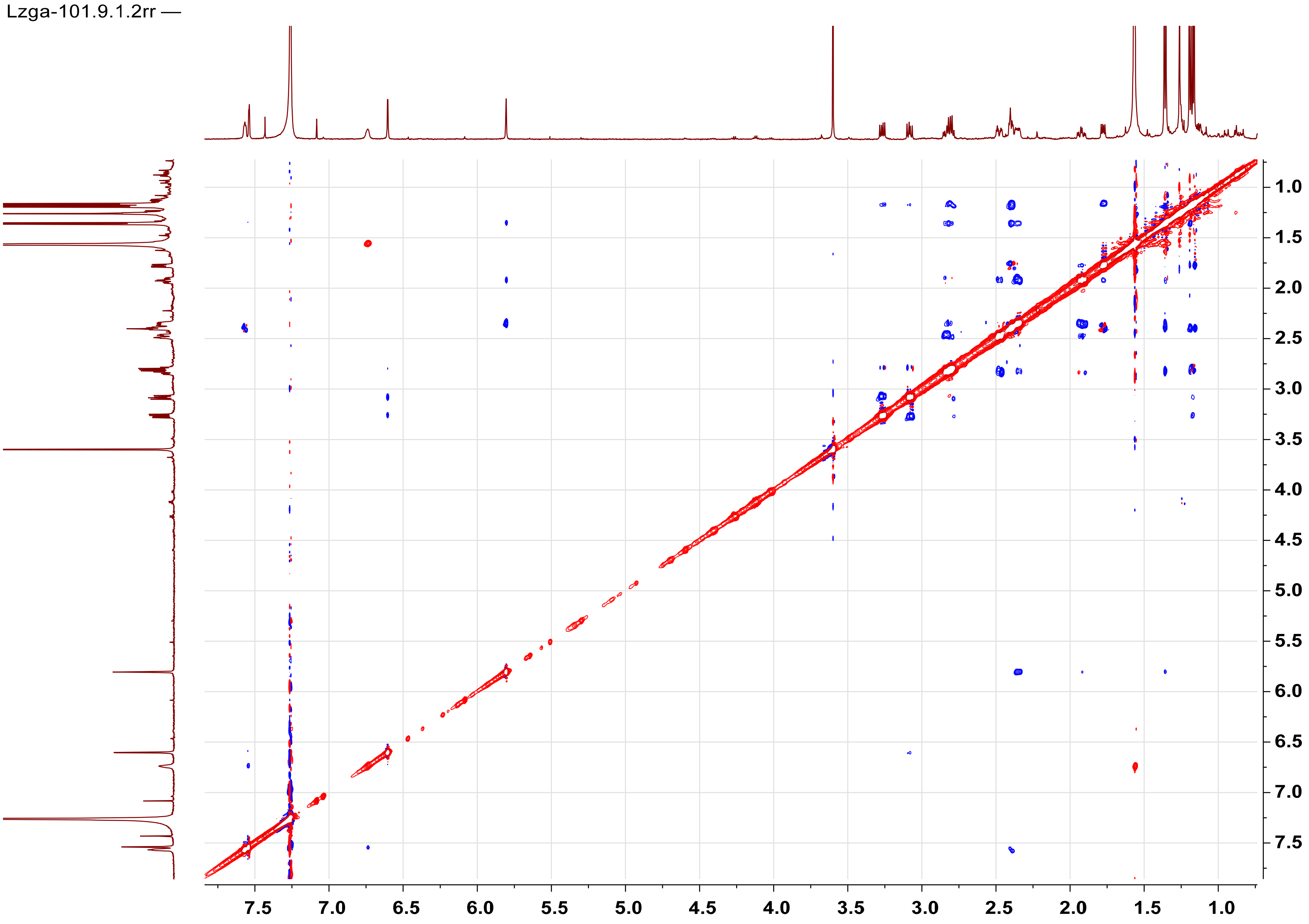

Figure S28. HRESIMS report of **2**.

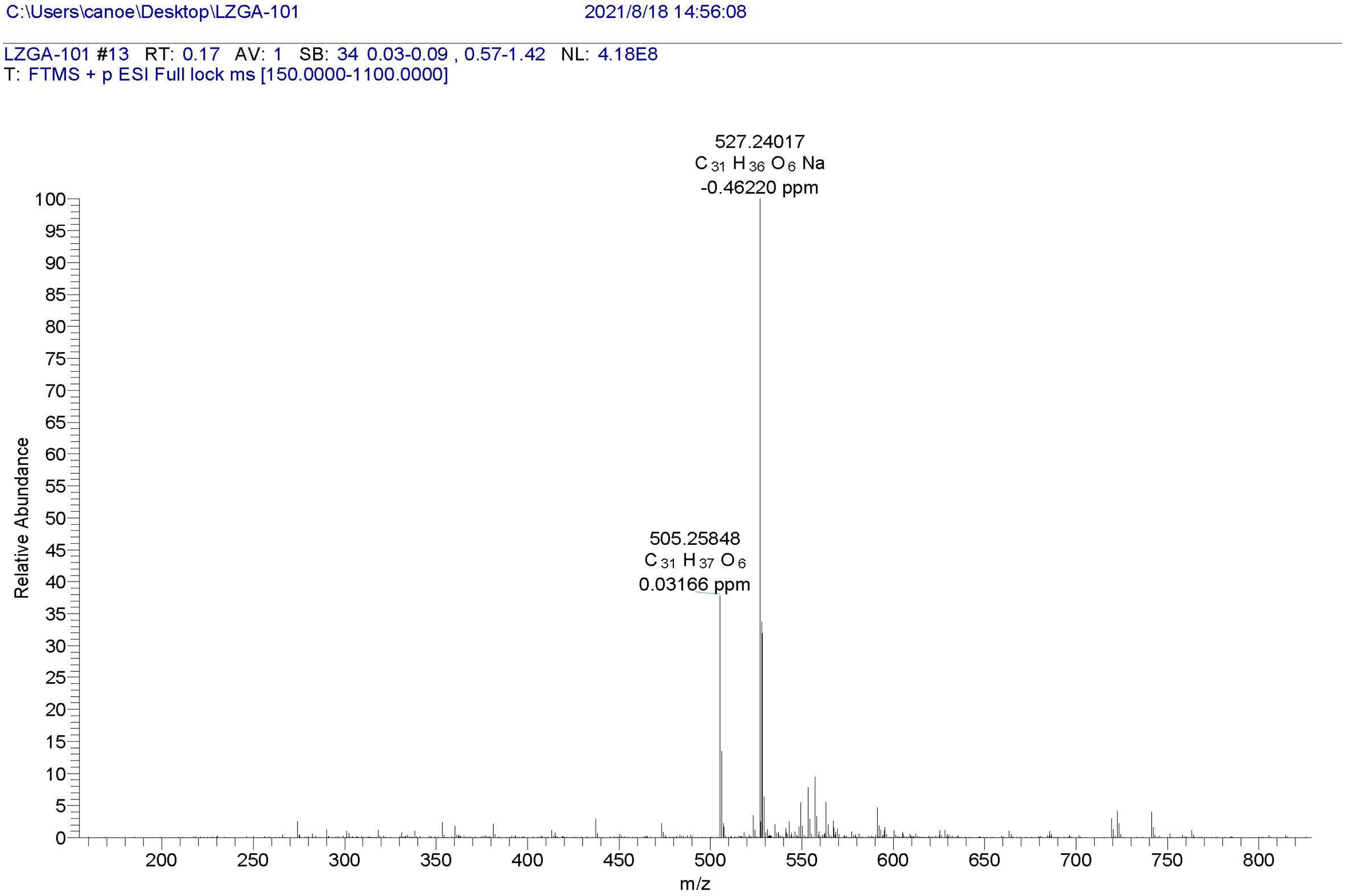

Figure S29. ^{1}H NMR spectrum of (*S*)-PGME-**1** (600 MHz, $CDCl_3$).

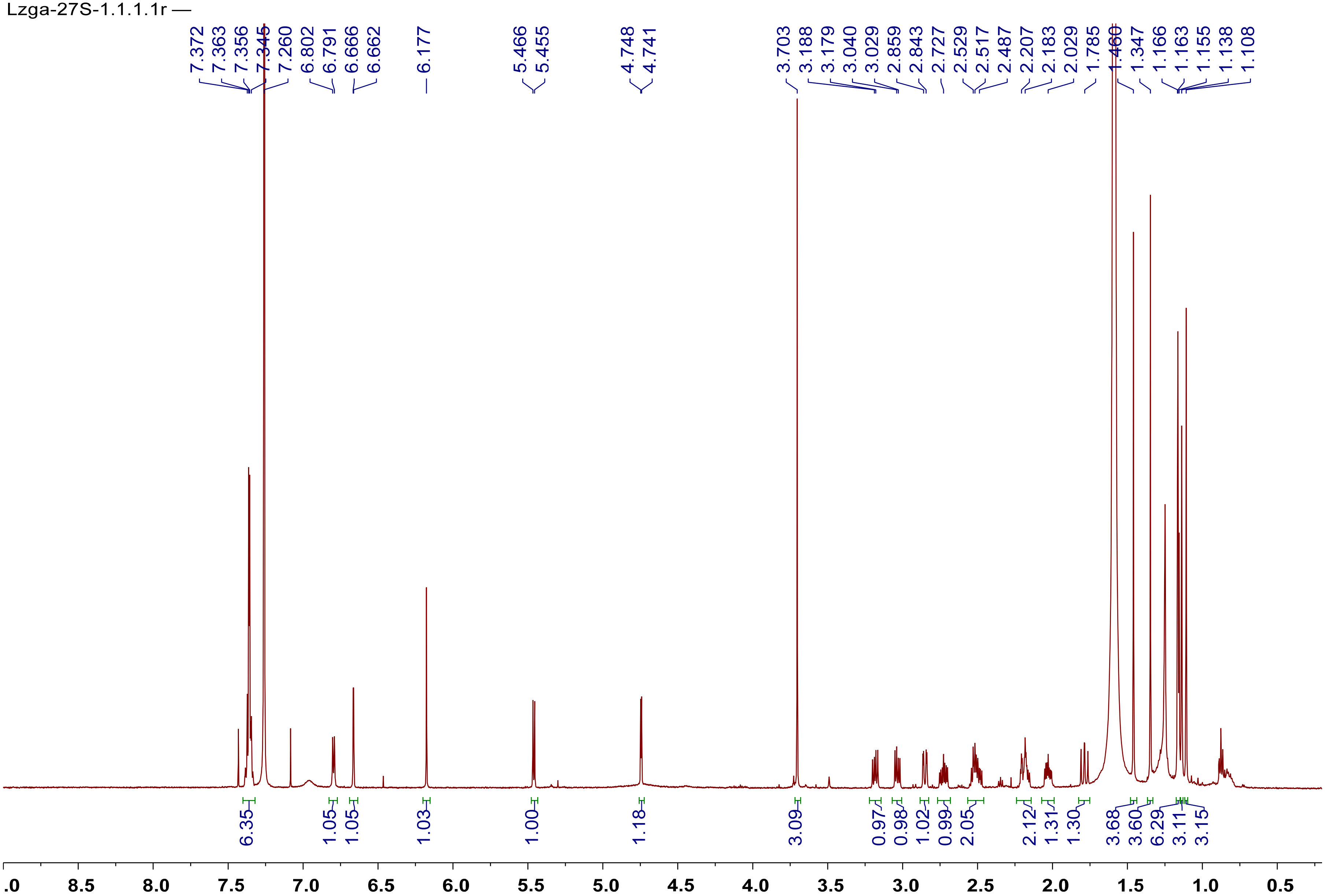

Figure S30. ^{1}H NMR spectrum of (*R*)-PGME-**1** (**1a**) (600 MHz, $CDCl_3$).

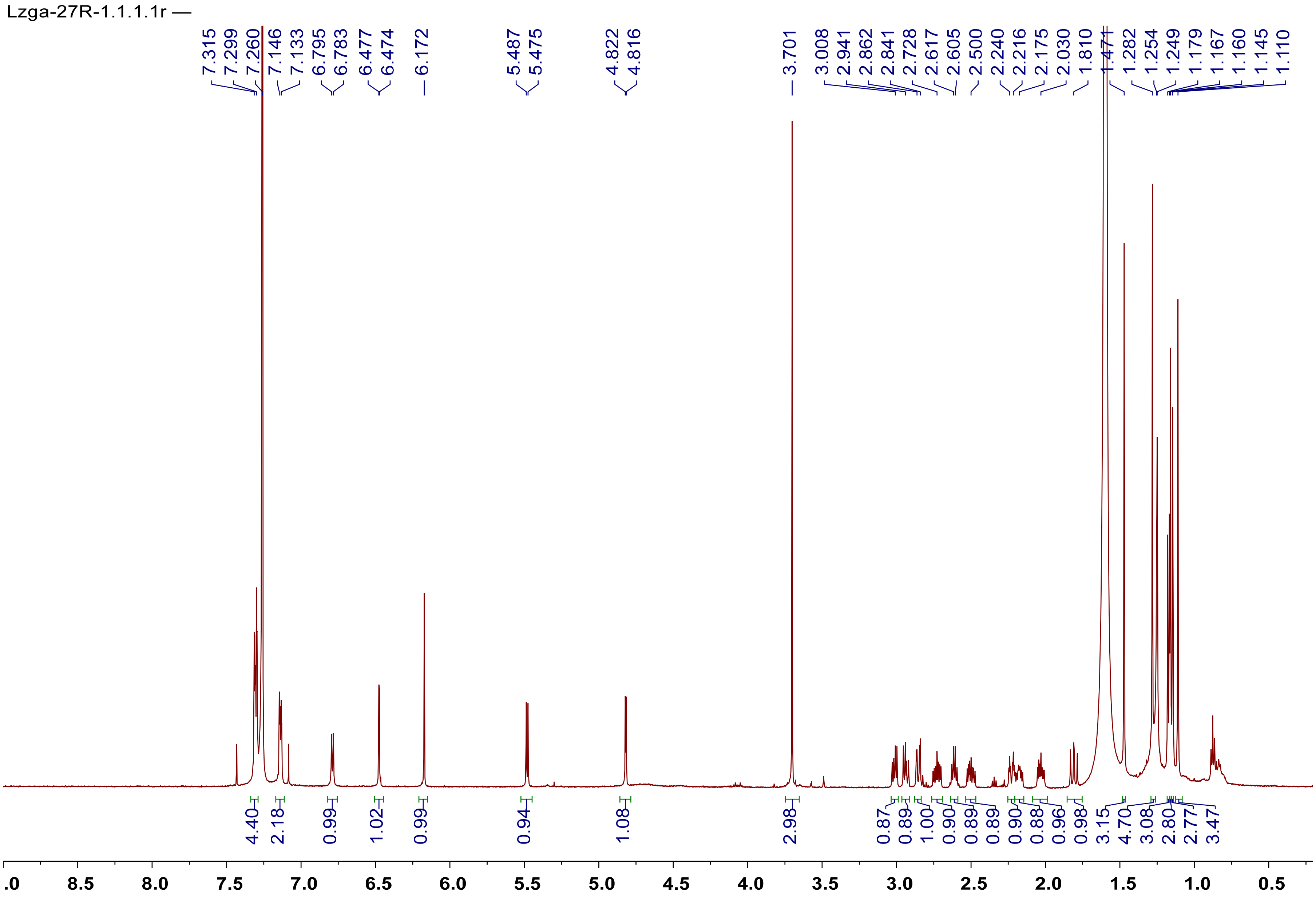

Figure S31. Comparison of ^{1}H NMR spectra of (*S*)- and (*R*)-PGME-**1** (**1a**) (600 MHz, $CDCl_3$).

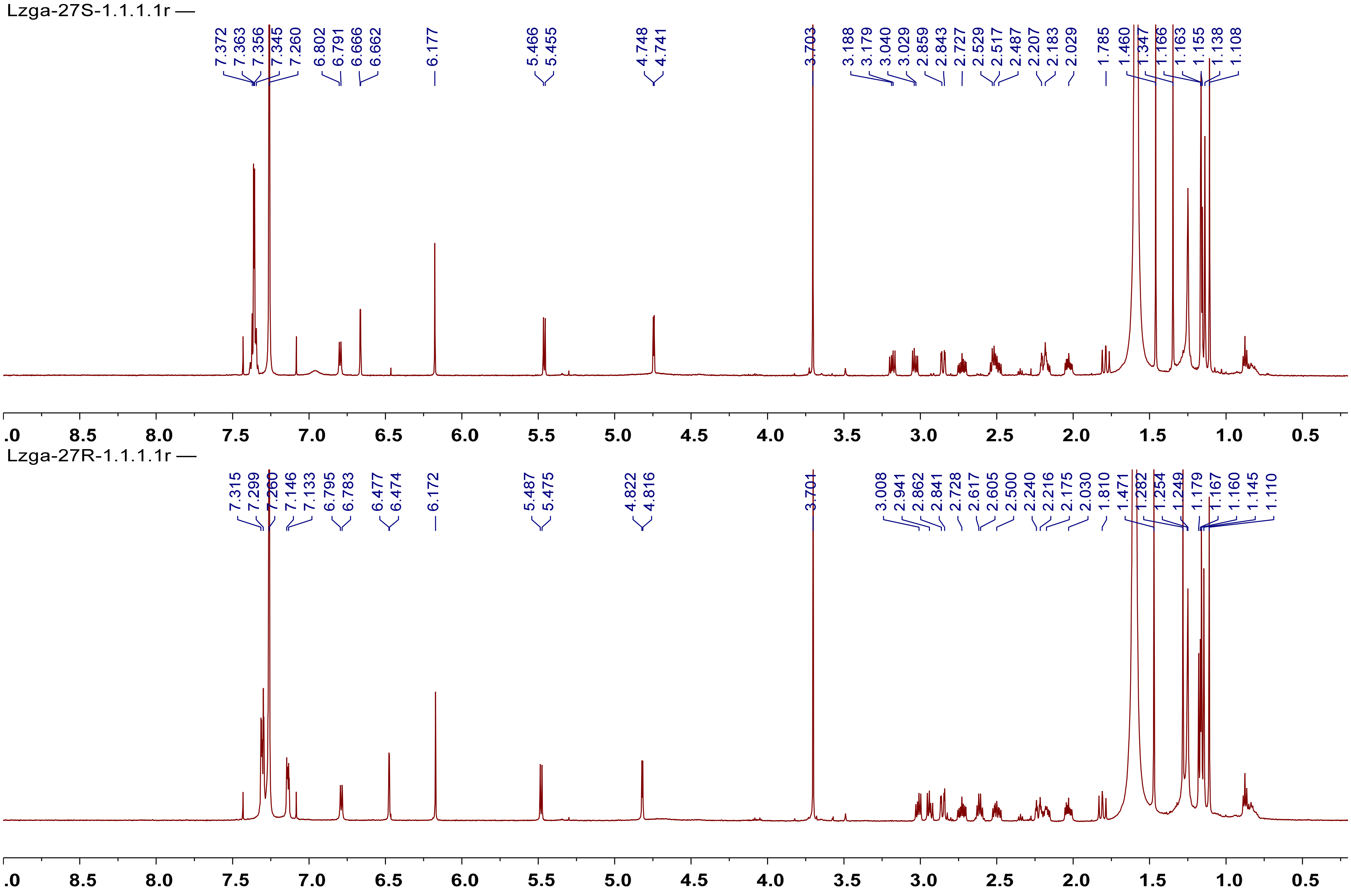

Figure S32. Enlarged comparison of ^{1}H NMR spectra of (*S*)- and (*R*)-PGME-**1** (**1a**) (600 MHz, $CDCl_3$).

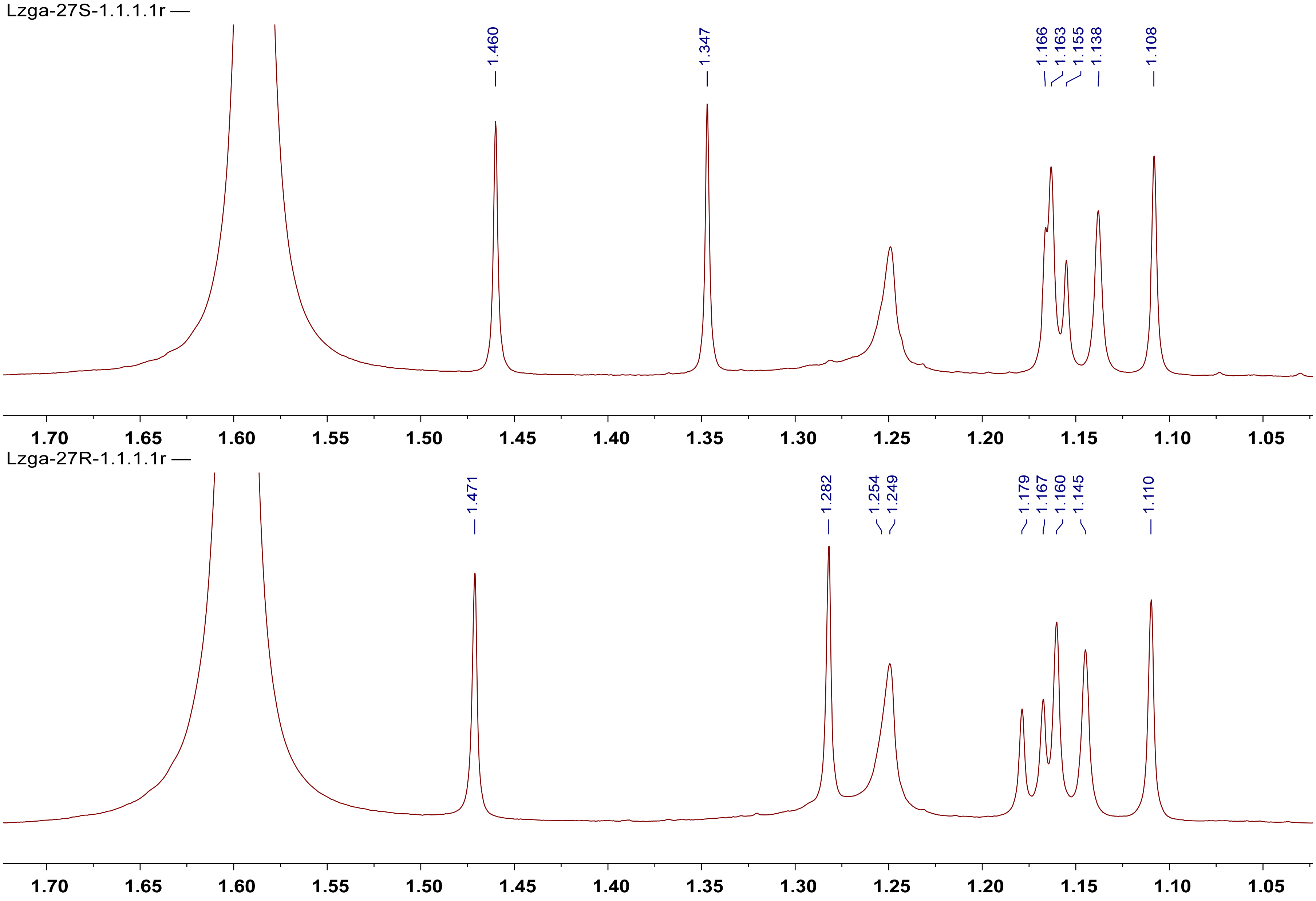

Figure S33. ^{1}H-^{1}H COSY spectrum of (*S*)-PGME-**1** (**1a**).

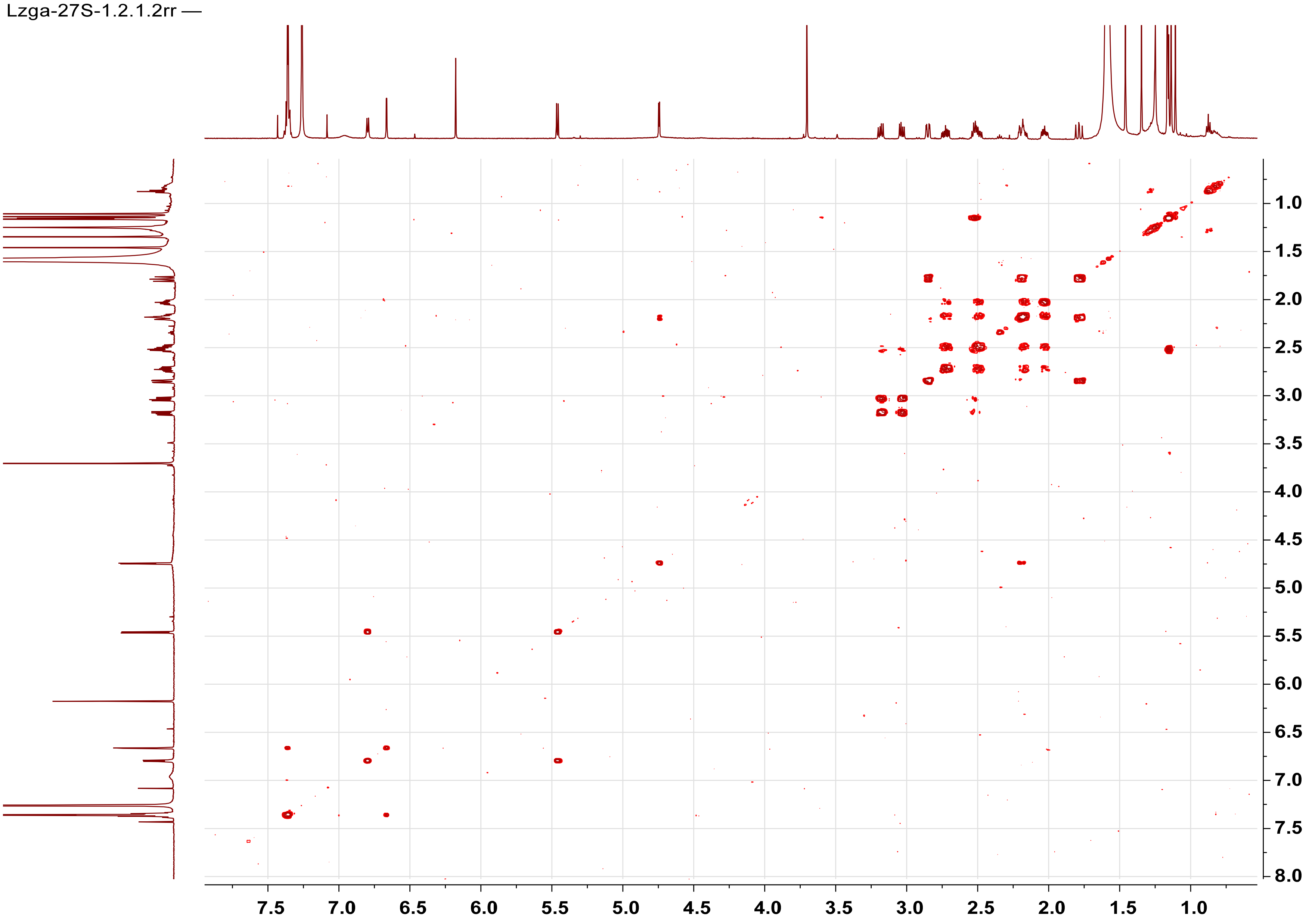

Figure S34. ^{1}H-^{1}H COSY spectrum of (*R*)-PGME-**1** (**1a**).

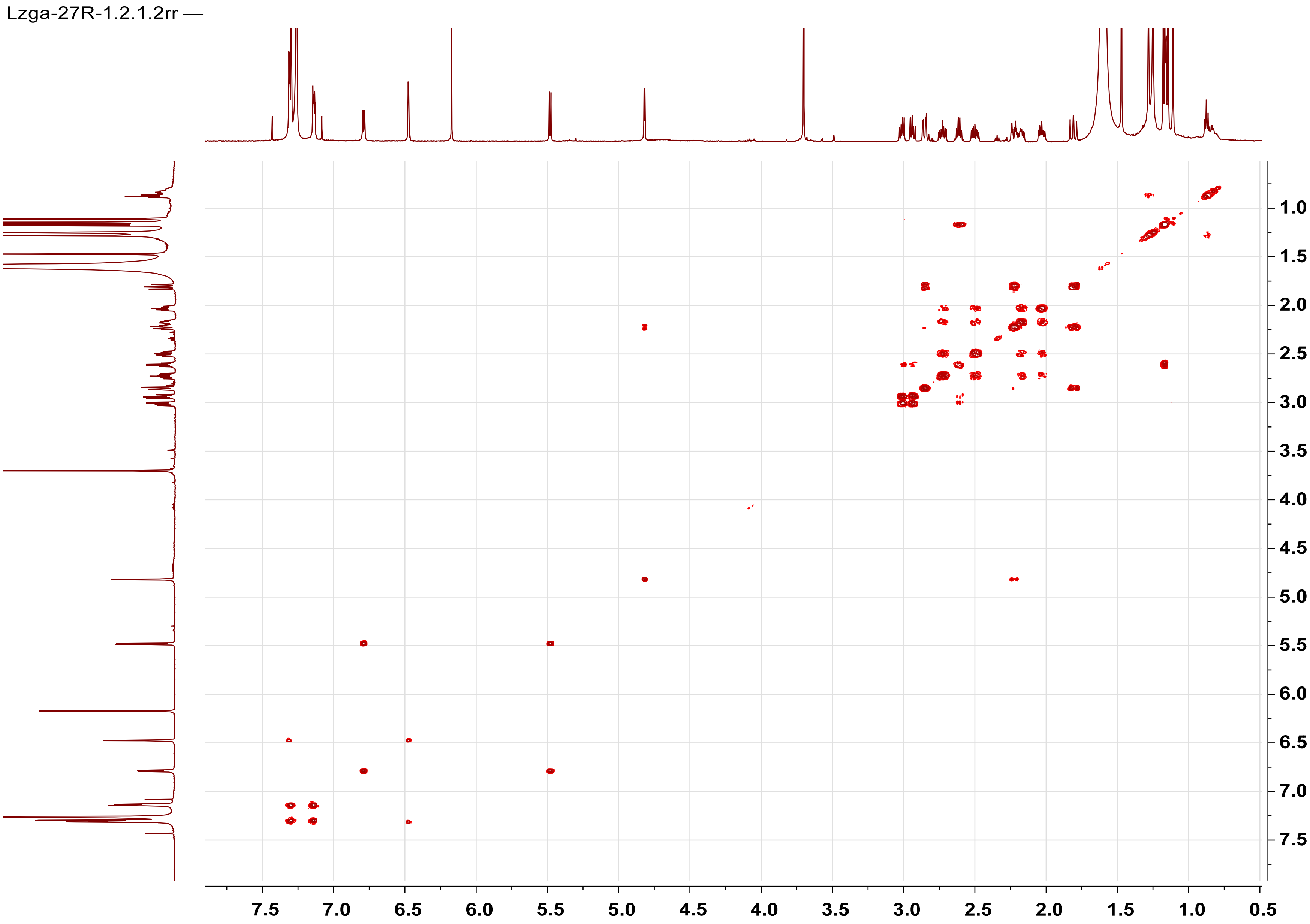

Figure S35. HRESIMS report of (*S*)-PGME-**1** (**1a**).

D:\spectrum\...\Lzga-27S-1\lzga-27S(1)　　2021/7/7 9:53:14

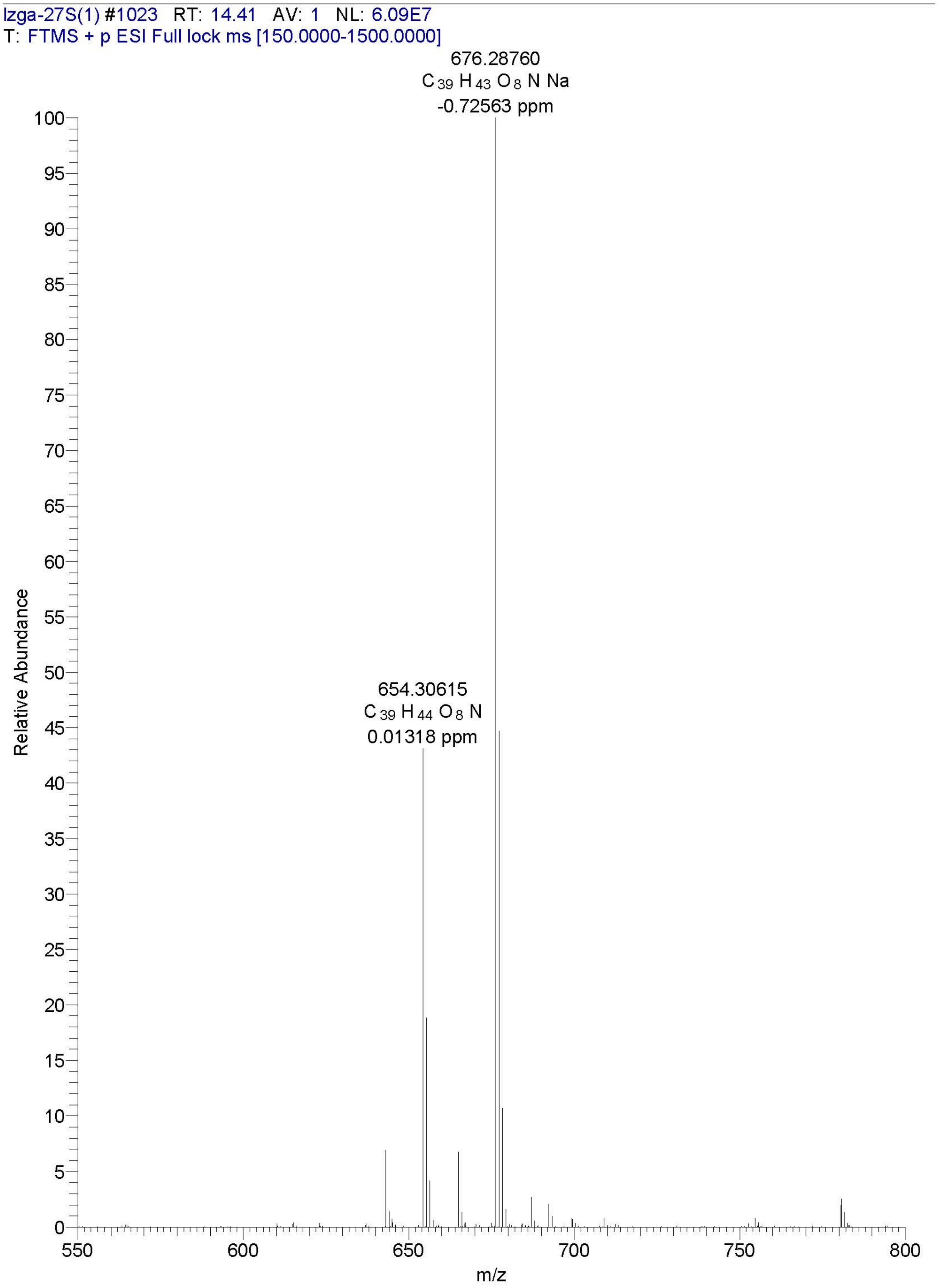

Figure S36. HRESIMS report of (*R*)-PGME-**1** (**1b**).

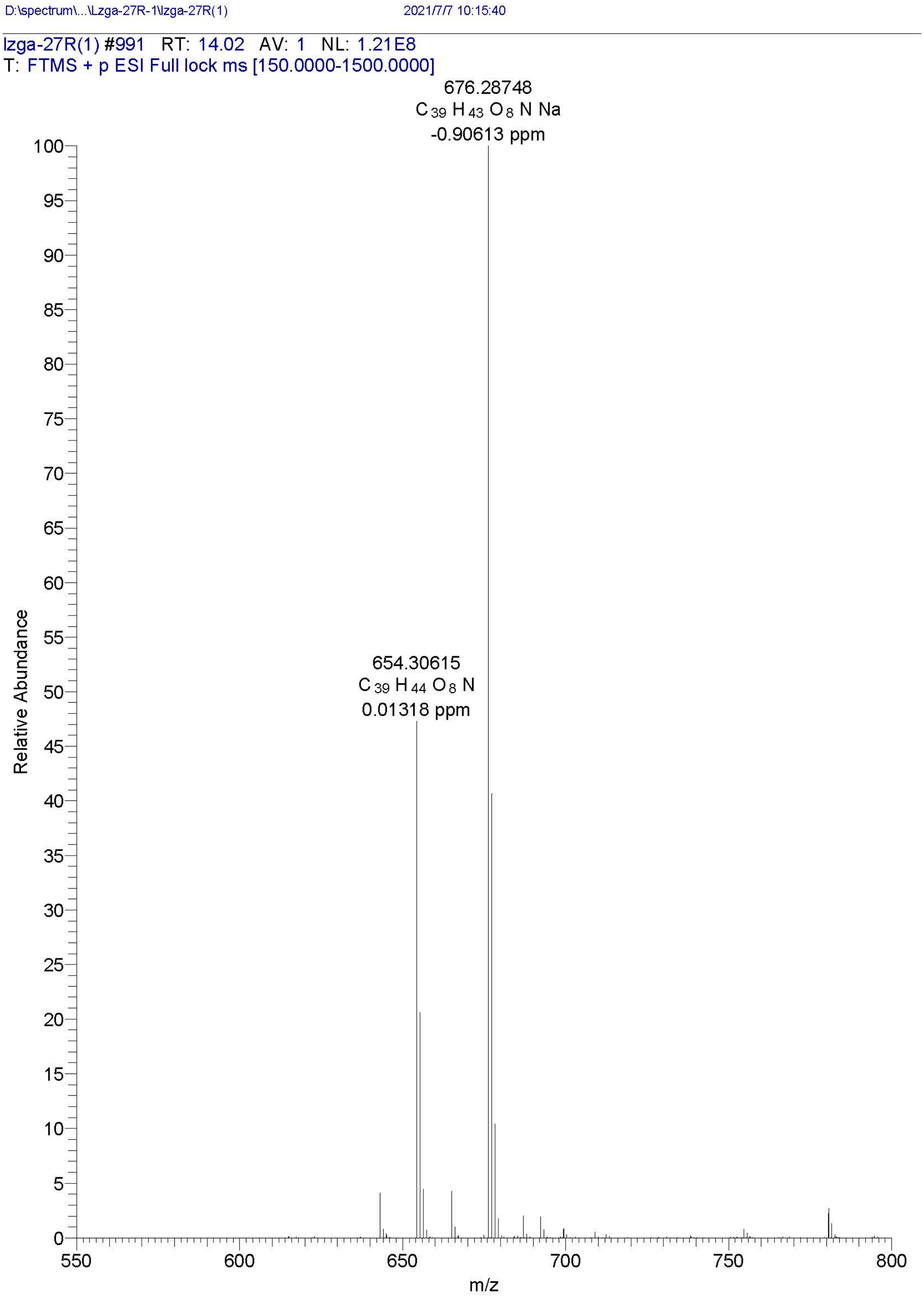

3. Calculation details.

Table S2. Conformational analysis of the M06-2X-D3/Def2-SVP optimized conformers of **1**.

Conformer No.	E (Hartree)	ΔG (kcal/mol)	Population (%)
**1a**	−1689.671274	1.18	6.8
**1b**	−1689.670256	1.82	2.3
**1c**	−1689.673153	0.00	49.8
**1d**	−1689.672969	0.12	41.0

Table S3. Calculated ^{13}C NMR analysis of **1**.

No.	Calcd Shielding Values	Calcd δ	Exptl δ	Corrected δ	Deviation
1	153.3	40.6	35.9	36.4	0.5
2	156.5	37.5	33.6	33.2	0.4
3	-27.5	221.5	216.6	218.1	1.6
4	142.4	51.5	46.1	47.3	1.2
5	149.0	44.9	40.8	40.7	0.2
6	165.9	28.0	22.9	23.7	0.7
7	132.5	61.4	58.9	57.2	1.7
8	128.4	65.5	62.6	61.4	1.2
9	16.5	177.4	167.6	173.9	6.3
10	147.3	46.7	40.8	42.4	1.6
11	59.4	134.6	130.1	130.8	0.7
12	-5.8	199.8	200.5	196.3	4.2
13	133.8	60.1	56.4	56.0	0.4
14	133.1	60.8	54.7	56.7	1.9
15	-11.2	205.1	201.1	201.7	0.6
16	66.1	127.8	123.8	124.0	0.2
17	30.0	163.9	155.7	160.3	4.6
18	157.3	36.7	31.2	32.4	1.2
19	164.6	29.3	25.3	25.0	0.3
20	81.0	112.9	111.1	109.0	2.0
21	27.6	166.3	161.6	162.7	1.1
22	75.6	118.3	117.8	114.4	3.3
23	42.6	151.3	143.7	147.6	3.9
24	151.8	42.1	36.4	37.9	1.4
25	146.7	47.3	40.8	43.1	2.3
26	18.1	175.8	181.6	172.3	9.4
27	175.8	18.1	17.7	13.7	3.9
28	161.3	32.7	28.9	28.4	0.5
29	169.4	24.5	21.7	20.2	1.5
30	166.5	27.5	23.8	23.1	0.7

Table S4. Calculated ^{1}H NMR analysis of **1**.

No.	Calcd Shielding Values	Calcd δ	Exptl δ	Corrected δ	Deviation
1a	29.330	2.185	2.19	2.23	0.04
1b	29.540	1.975	2.02	2.03	0.01
2a	29.382	2.134	2.50	2.18	0.32
2b	28.595	2.920	2.74	2.95	0.21
5	28.713	2.802	2.86	2.84	0.02
6a	29.766	1.750	1.80	1.81	0.01
6b	29.320	2.195	2.23	2.24	0.01
7	26.857	4.658	4.79	4.65	0.14
11	25.367	6.148	6.20	6.10	0.10
18	29.959	1.556	1.50	1.62	0.12
19	30.425	1.090	1.15	1.16	0.01
20	23.905	7.610	7.35	7.53	0.18
22	24.835	6.681	6.54	6.62	0.08
24a	28.149	3.367	3.29	3.39	0.10
24b	29.170	2.346	2.74	2.39	0.35
25	29.006	2.509	2.64	2.55	0.09
27	30.402	1.113	1.18	1.19	0.01
28	30.359	1.156	1.15	1.23	0.08
29	30.433	1.082	1.10	1.16	0.06
30	30.129	1.386	1.34	1.45	0.11

Table S5. Conformational analysis of the M06-2X-D3/Def2-SVP optimized conformers of **2**.

Conformer No.	E (Hartree)	ΔG (kcal/mol)	Population (%)
**2a**	−1651.893922	0.00	26.0
**2b**	−1651.893824	0.06	23.4
**2c**	−1651.892838	0.68	8.2
**2d**	−1651.893795	0.08	22.7
**2e**	−1651.891613	1.45	2.3
**2f**	−1651.89348	0.28	16.3
**2g**	−1651.891019	1.82	1.2

Table S6. Calculated ^{13}C NMR analysis of **2**.

No.	Calcd Shielding Values	Calcd δ	Exptl δ	Corrected δ	Deviation
1	153.9	40.1	35.8	35.7	0.1
2	155.2	38.7	34.5	34.3	0.2
3	-24.9	218.8	214.8	215.0	0.2
4	139.8	54.1	47.5	49.8	2.3
5	140.1	53.9	49.7	49.5	0.2
6	164.3	29.6	24.1	25.2	1.1
7	49.3	144.6	135.0	140.5	5.5
8	52.6	141.3	134.9	137.3	2.4
9	24.3	169.7	163.2	165.7	2.5
10	150.0	43.9	38.8	39.6	0.8
11	73.7	120.2	117.6	116.1	1.5
12	-7.1	201.1	200.5	197.2	3.3
13	135.7	58.3	54.4	54.0	0.4
14	133.8	60.1	56.0	55.8	0.2
15	-12.0	205.9	203.3	202.1	1.2
16	65.0	128.9	124.8	124.8	0.0
17	30.8	163.1	156.5	159.1	2.6
18	157.1	36.8	31.9	32.5	0.6
19	168.3	25.6	21.7	21.2	0.5
20	81.6	112.3	111.3	108.2	3.1
21	28.4	165.5	161.0	161.5	0.5
22	75.3	118.6	116.9	114.5	2.4
23	43.4	150.6	143.9	146.5	2.6
24	154.3	39.6	35.9	35.3	0.6
25	147.0	46.9	40.6	42.6	2.0
26	16.9	177.0	176.7	173.1	3.6
27	172.6	21.3	17.2	16.9	0.3
28	166.0	27.9	22.7	23.5	0.8
29	167.3	26.6	25.5	22.2	3.3
30	160.6	33.3	29.0	28.9	0.1
31	140.8	53.2	51.8	48.8	3.0

Table S7. Calculated ^{1}H NMR analysis of **2**.

No.	Calcd Shielding Values	Calcd δ	Exptl δ	Corrected δ	Deviation
1a	29.799	1.72	1.91	1.77	0.15
1b	29.290	2.23	2.35	2.26	0.09
2a	28.518	3.00	2.82	3.01	0.19
2b	29.311	2.20	2.47	2.24	0.23
5	30.199	1.32	1.78	1.39	0.41
6a	28.982	2.53	2.40	2.56	0.16
6b	29.112	2.40	2.40	2.44	0.03
7	23.499	8.02	7.56	7.86	0.34
11	25.853	5.66	5.80	5.58	0.20
18	30.186	1.33	1.35	1.40	0.04
19	30.029	1.49	1.36	1.55	0.18
20	24.002	7.51	7.54	7.37	0.13
22	24.734	6.78	6.60	6.66	0.09
24a	28.613	2.90	3.07	2.92	0.15
24b	28.254	3.26	3.26	3.26	0.01
25	28.980	2.54	2.80	2.56	0.24
27	30.425	1.09	1.17	1.17	0.02
27	30.263	1.25	1.17	1.32	0.14
27	30.081	1.43	1.17	1.50	0.32
28	30.419	1.10	1.16	1.17	0.00
29	30.280	1.24	1.19	1.31	0.10
30	30.274	1.24	1.26	1.31	0.04
31	27.946	3.57	3.59	3.56	0.02

Table S8. Standard orientations of conformers.

1a			
C	5.69175	-0.58793	-1.24375
C	6.11312	0.2455	-0.04748
C	5.12997	1.34962	0.36403
C	3.68615	0.92617	-0.02925
C	3.45739	-0.61269	0.03895
C	4.31076	-1.25832	-1.07958
C	2.60491	1.71229	0.71113
C	1.25994	1.45006	0.07433
C	0.95155	0.08044	-0.39485
C	1.98443	-0.99815	-0.16649
C	-0.43755	-0.46153	-0.65368
C	-0.76211	-1.62178	0.31431
C	0.19331	-2.75601	0.00767
C	1.60675	-2.29353	-0.08407
C	-1.6885	0.41198	-0.50711
C	-2.79191	-0.53765	-0.23713
C	-2.25054	-1.78409	0.11643
C	-4.17915	-0.35504	-0.36868
C	-4.99222	-1.4639	-0.1553
C	-4.4437	-2.71441	0.17935
C	-3.05774	-2.88684	0.3306
O	-5.30553	-3.72966	0.34897
O	-0.12613	-3.9196	-0.0518
C	3.88973	-1.18133	1.40731
C	-0.55048	-1.21175	1.79392
C	-0.46705	-0.91636	-2.13648
O	-1.74239	1.61657	-0.65824
C	5.53273	2.58111	-0.47567
C	5.3246	1.70997	1.83977
O	7.17443	0.09433	0.50273
O	1.3174	1.1129	-1.29323
C	-4.73339	0.99976	-0.70996
C	-4.68391	1.9614	0.50252
C	-5.82105	1.71106	1.47854
H	3.5694	1.19075	-1.08994
C	-4.6842	3.40528	0.00437
O	-5.5857	4.17129	0.18697
O	-3.59314	3.75642	-0.68192
H	6.4867	-1.31612	-1.44357
H	5.65641	0.1048	-2.09983
H	3.75909	-1.19278	-2.02837
H	4.43463	-2.329	-0.86024
H	2.56334	1.4474	1.78046
H	2.82087	2.79083	0.66227
H	0.41676	2.0934	0.34267
H	2.34528	-3.09049	0.02845
H	-6.0761	-1.39238	-0.25784
H	-2.62453	-3.85527	0.58806
H	-4.82729	-4.53884	0.56517
H	3.34064	-0.70472	2.2336
H	4.96443	-1.04515	1.58773
H	3.68945	-2.25891	1.45432
H	-1.14415	-0.31559	2.02736
H	-0.89528	-2.02388	2.44909
H	0.50404	-1.00716	2.01908
H	0.39021	-1.56417	-2.36259
H	-1.39132	-1.45923	-2.37233
H	-0.39596	-0.02462	-2.77263
H	5.51983	2.36475	-1.55404
H	4.83142	3.40867	-0.29144
H	6.54216	2.91585	-0.19878
H	4.96633	0.92897	2.52191
H	6.39493	1.85631	2.03375
H	4.79643	2.64351	2.08019
H	-4.14653	1.41958	-1.54038
H	-5.77438	0.91167	-1.05415
H	-3.7134	1.80907	1.00515
H	-6.78256	1.94056	0.99899
H	-5.8242	0.66205	1.80833
H	-5.73191	2.35832	2.36057
H	-2.93773	3.03378	-0.6913

1b			
C	5.69174	-0.58793	-1.24376
C	6.11312	0.24551	-0.04748
C	5.12997	1.34962	0.36403
C	3.68615	0.92617	-0.02925
C	3.45739	-0.61269	0.03895
C	4.31076	-1.25832	-1.07958
C	2.60491	1.71228	0.71113
C	1.25994	1.45005	0.07434
C	0.95155	0.08044	-0.39485
C	1.98443	-0.99815	-0.16648
C	-0.4375	-0.46153	-0.65368
C	-0.7621	-1.62178	0.31431
C	0.19331	-2.75601	0.00768
C	1.60675	-2.29353	-0.08406
C	-1.6885	0.41198	-0.50712
C	-2.7919	-0.53765	-0.23713
C	-2.2505	-1.78409	0.11643
C	-4.1792	-0.35504	-0.36868
C	-4.9922	-1.4639	-0.1553
C	-4.4437	-2.7144	0.17935
C	-3.0578	-2.88684	0.3306
O	-5.3055	-3.72966	0.34898
O	-0.1261	-3.9196	-0.0518
C	3.88973	-1.18134	1.40731
C	-0.5505	-1.21175	1.79392
C	-0.4671	-0.91637	-2.13648
O	-1.7424	1.61657	-0.65824
C	5.53272	2.58111	-0.47566
C	5.3246	1.70996	1.83977
O	7.17443	0.09434	0.50272
O	1.3174	1.1129	-1.29323
C	-4.7334	0.99976	-0.70996
C	-4.6839	1.9614	0.50252
C	-5.8211	1.71107	1.47854
H	3.56939	1.19075	-1.08994
C	-4.6842	3.40528	0.00437
O	-5.5857	4.1713	0.18697
O	-3.5931	3.75641	-0.68193
H	6.48669	-1.31611	-1.44358
H	5.6564	0.10482	-2.09984
H	3.75909	-1.19278	-2.02837
H	4.43463	-2.32899	-0.86024
H	2.56334	1.44739	1.78047
H	2.82087	2.79083	0.66228
H	0.41676	2.09339	0.34267
H	2.34528	-3.09049	0.02845
H	-6.0761	-1.39238	-0.25784
H	-2.6245	-3.85527	0.58806
H	-4.8273	-4.53884	0.56518
H	3.34065	-0.70473	2.2336
H	4.96444	-1.04515	1.58773
H	3.68945	-2.25892	1.45432
H	-1.1442	-0.31559	2.02736
H	-0.8953	-2.02388	2.4491
H	0.50404	-1.00715	2.01908
H	0.39021	-1.56418	-2.36259
H	-1.3913	-1.45924	-2.37233
H	-0.396	-0.02463	-2.77263
H	5.51982	2.36476	-1.55403
H	4.83142	3.40868	-0.29143
H	6.54215	2.91586	-0.19877
H	4.96633	0.92895	2.52191
H	6.39493	1.85631	2.03375
H	4.79643	2.64349	2.08021
H	-4.1465	1.41958	-1.54038
H	-5.7744	0.91167	-1.05415
H	-3.7134	1.80907	1.00515
H	-6.7826	1.94057	0.99898
H	-5.8242	0.66205	1.80832
H	-5.7319	2.35833	2.36056

H	-2.9377	3.03377	-0.6913

1c			
C	5.69359	-0.58689	-1.2437
C	6.11425	0.24674	-0.04737
C	5.1296	1.34913	0.3653
C	3.6862	0.92411	-0.02776
C	3.45935	-0.61502	0.03927
C	4.31319	-1.25861	-1.07996
C	2.60431	1.70817	0.71385
C	1.25926	1.44505	0.07754
C	0.95251	0.07575	-0.39317
C	1.98665	-1.00176	-0.16629
C	-0.43628	-0.46746	-0.65186
C	-0.76131	-1.63011	0.31412
C	0.19562	-2.76216	0.0009
C	1.60961	-2.29695	-0.08539
C	-1.68548	0.40667	-0.5053
C	-2.7895	-0.54187	-0.2354
C	-2.2508	-1.79313	0.11895
C	-4.17223	-0.35345	-0.36572
C	-4.98792	-1.46302	-0.14694
C	-4.44454	-2.71422	0.19039
C	-3.05759	-2.89253	0.33656
O	-5.2233	-3.78932	0.39049
O	-0.12129	-3.92457	-0.07219
C	3.89278	-1.1844	1.4069
C	-0.54922	-1.22363	1.79433
C	-0.46709	-0.92084	-2.13528
O	-1.74016	1.61162	-0.65575
C	5.53052	2.58183	-0.47347
C	5.32425	1.70843	1.8413
O	7.17636	0.09748	0.50187
O	1.31603	1.11008	-1.2907
C	-4.72518	1.00131	-0.70909
C	-4.67545	1.96577	0.50099
C	-5.81334	1.7199	1.4772
H	3.56857	1.18947	-1.08819
C	-4.67464	3.40779	-0.00253
O	-5.57763	4.17343	0.17479
O	-3.58251	3.75611	-0.6876
H	6.48935	-1.31415	-1.44372
H	5.65772	0.10614	-2.09955
H	3.76128	-1.19265	-2.02856
H	4.43797	-2.3294	-0.86178
H	2.56355	1.44188	1.78288
H	2.81903	2.78704	0.66617
H	0.41591	2.08747	0.34747
H	2.34837	-3.09386	0.02638

H	-6.07203	-1.36212	-0.25215
H	-2.65209	-3.86919	0.59757
H	-6.15067	-3.55849	0.26238
H	3.34356	-0.70905	2.23382
H	4.9675	-1.04774	1.58713
H	3.69309	-2.26213	1.45298
H	-1.14476	-0.32946	2.03119
H	-0.89159	-2.03875	2.44699
H	0.50498	-1.01673	2.01898
H	0.39034	-1.56809	-2.36214
H	-1.39076	-1.46532	-2.37001
H	-0.39727	-0.02879	-2.77114
H	5.51762	2.36626	-1.552
H	4.82813	3.4083	-0.28842
H	6.5396	2.91768	-0.19659
H	4.96717	0.92625	2.5227
H	6.39445	1.85597	2.03508
H	4.79488	2.64103	2.08276
H	-4.13727	1.41872	-1.53984
H	-5.76622	0.91553	-1.05464
H	-3.70563	1.81336	1.00438
H	-6.77482	1.94642	0.99596
H	-5.81461	0.67293	1.81384
H	-5.7255	2.37187	2.35583
H	-2.92655	3.03357	-0.69316

1d			
C	-5.77441	0.50734	-1.23485
C	-6.17662	-0.36414	-0.05926
C	-5.17126	-1.46023	0.31922
C	-3.73789	-0.99987	-0.06971
C	-3.53677	0.54037	0.04114
C	-4.4067	1.20063	-1.05573
C	-2.63738	-1.78755	0.6399
C	-1.30222	-1.48264	0.00081
C	-1.02119	-0.09349	-0.4265
C	-2.07159	0.95811	-0.15878
C	0.35828	0.47721	-0.67264
C	0.66716	1.61175	0.3302
C	-0.30816	2.73864	0.06326
C	-1.71504	2.25617	-0.03682
C	1.62227	-0.38935	-0.54138
C	2.71606	0.5792	-0.25364
C	2.15263	1.80584	0.13222
C	4.10429	0.42991	-0.39814
C	4.89243	1.55589	-0.16339
C	4.32282	2.78474	0.20463
C	2.93541	2.9219	0.36958
O	5.16412	3.8177	0.39353

O	-0.01203	3.91	0.03739
C	-3.9743	1.06311	1.4262
C	0.46752	1.15192	1.79676
C	0.37906	0.97813	-2.13845
O	1.69934	-1.58462	-0.69645
C	-5.55689	-2.6762	-0.55055
C	-5.35093	-1.86301	1.78571
O	-7.23901	-0.24748	0.49754
O	-1.37816	-1.1034	-1.35479
C	4.72356	-0.88791	-0.77824
C	4.69906	-1.9048	0.36888
C	5.39891	-1.41426	1.63946
H	-3.62255	-1.23134	-1.13826
C	5.31822	-3.20274	-0.10386
O	5.83382	-3.38576	-1.17211
O	5.24054	-4.16611	0.8286
H	-6.58405	1.22419	-1.41607
H	-5.72641	-0.16381	-2.10745
H	-3.85688	1.17323	-2.00743
H	-4.55106	2.26199	-0.80548
H	-2.59227	-1.55289	1.71612
H	-2.83601	-2.8678	0.56156
H	-0.44376	-2.11748	0.23972
H	-2.4656	3.0374	0.10425
H	5.97684	1.51019	-0.27491
H	2.48156	3.87364	0.65283
H	4.66514	4.6074	0.63252
H	-3.41175	0.57583	2.23708
H	-5.04546	0.90183	1.60793
H	-3.794	2.14257	1.50106
H	1.07262	0.25542	1.99703
H	0.807	1.94636	2.47613
H	-0.58334	0.92744	2.02098
H	-0.4783	1.63406	-2.34192
H	1.30293	1.52709	-2.3616
H	0.30655	0.10665	-2.80191
H	-5.55738	-2.43027	-1.62269
H	-4.83727	-3.49382	-0.39475
H	-6.55718	-3.03839	-0.2749
H	-4.99517	-1.09694	2.48567
H	-6.4181	-2.02659	1.98358
H	-4.81026	-2.79661	1.99681
H	4.18381	-1.32401	-1.63104
H	5.76476	-0.73353	-1.0968
H	3.64946	-2.14271	0.60552
H	6.45171	-1.16593	1.43314
H	4.90683	-0.51063	2.02455
H	5.37091	-2.18694	2.41798
H	5.65968	-4.95613	0.45461

2a			
C	6.11383	0.89943	0.89185
C	6.2904	-0.59822	1.00703
C	5.4691	-1.45891	0.03501
C	4.00206	-0.93312	-0.04124
C	3.77724	0.60699	-0.07569
C	4.63676	1.27385	1.00769
C	3.18418	-1.60479	-1.14498
C	1.74149	-1.21221	-1.05983
C	1.3269	-0.09813	-0.43116
C	2.2863	0.86945	0.15778
C	-0.116	0.20483	-0.12021
C	-0.39908	1.71763	-0.27672
C	0.43239	2.45478	0.75162
C	1.84766	1.99215	0.78052
C	-1.28295	-0.41263	-0.9096
C	-2.40229	0.55359	-0.75438
C	-1.90815	1.76331	-0.24325
C	-3.77533	0.36466	-0.97867
C	-4.63246	1.40717	-0.63449
C	-4.13388	2.60642	-0.09976
C	-2.75676	2.80477	0.09042
O	-5.03357	3.55555	0.21943
O	0.03468	3.38826	1.41009
C	4.07404	1.25048	-1.44847
C	0.03	2.23555	-1.67264
C	-0.38031	-0.27016	1.3374
O	-1.30431	-1.50156	-1.43691
C	5.47093	-2.89853	0.55846
C	6.22469	-1.44133	-1.30924
O	7.04489	-1.08734	1.80939
H	0.98474	-1.88299	-1.47315
C	-4.28963	-0.94524	-1.51704
C	-4.31076	-2.04147	-0.44797
C	-4.49204	-3.43352	-1.06132
H	3.54507	-1.25042	0.9145
C	-5.42045	-1.7795	0.54994
O	-6.36691	-1.05989	0.3715
O	-5.2498	-2.48865	1.67255
H	6.51573	1.23285	-0.07862
H	6.71322	1.36885	1.68183
H	4.262	0.97027	1.99936
H	4.53987	2.36728	0.95112
H	3.58846	-1.35121	-2.14114
H	3.25381	-2.69984	-1.06795
H	2.53202	2.68195	1.27513
H	-5.71136	1.29453	-0.74675
H	-2.36214	3.73647	0.50117
H	-4.57904	4.32468	0.58173
H	3.42049	0.84521	-2.23397
H	5.11432	1.11177	-1.76673
H	3.88176	2.33155	-1.38509
H	-0.45222	1.63732	-2.4591
H	-0.29851	3.27786	-1.7891
H	1.11751	2.19188	-1.81213
H	0.40282	0.09288	2.01596
H	-1.35471	0.08194	1.70394
H	-0.36939	-1.36875	1.3573
H	4.95548	-2.96657	1.52716
H	4.98166	-3.58007	-0.15055
H	6.50269	-3.23838	0.71165
H	6.32071	-0.44042	-1.74775
H	7.2385	-1.84062	-1.15919
H	5.72011	-2.08687	-2.04212
H	-5.30893	-0.81383	-1.90611
H	-3.63595	-1.2814	-2.33267
H	-3.36284	-2.0381	0.11252
H	-5.41552	-3.47542	-1.6581
H	-3.63965	-3.65561	-1.71745
H	-4.54416	-4.20232	-0.27916
C	-6.27081	-2.35324	2.64915
H	-5.9768	-2.98414	3.49366
H	-7.23839	-2.68016	2.24431
H	-6.36366	-1.30558	2.96585

2b			
C	6.12503	0.9006	0.8865
C	6.30225	-0.59685	1.00275
C	5.4785	-1.45887	0.03393
C	4.01131	-0.93315	-0.03982
C	3.78647	0.60687	-0.07587
C	4.64813	1.27496	1.00503
C	3.19102	-1.60613	-1.14096
C	1.74833	-1.21361	-1.05303
C	1.33524	-0.09865	-0.42509
C	2.29601	0.86965	0.16044
C	-0.10726	0.20568	-0.1128
C	-0.3919	1.71881	-0.27109
C	0.44077	2.45353	0.75916
C	1.85789	1.99251	0.78214
C	-1.27291	-0.41613	-0.89881
C	-2.39184	0.55073	-0.7516
C	-1.90194	1.76626	-0.24003
C	-3.7595	0.35688	-0.98146
C	-4.61819	1.40251	-0.64175
C	-4.12673	2.60301	-0.10649
C	-2.75014	2.80666	0.0895
O	-4.95367	3.60788	0.23711
O	0.04432	3.37962	1.42681
C	4.08068	1.24852	-1.45003
C	0.03722	2.2358	-1.66708
C	-0.37126	-0.26582	1.34614
O	-1.29669	-1.50981	-1.41695
C	5.48151	-2.89773	0.55946
C	6.231	-1.44334	-1.31205
O	7.05925	-1.0851	1.80337
H	0.9911	-1.88536	-1.46369
C	-4.27569	-0.95344	-1.51703
C	-4.32371	-2.04072	-0.43989
C	-4.51368	-3.43552	-1.04435
H	3.55634	-1.24911	0.91731
C	-5.43914	-1.75691	0.54587
O	-6.35981	-1.00324	0.36572
O	-5.30682	-2.48455	1.66042
H	6.52472	1.23302	-0.08525
H	6.72618	1.37092	1.67461
H	4.27535	0.97242	1.99775
H	4.55087	2.36829	0.94758
H	3.59324	-1.35339	-2.13819
H	3.26111	-2.70111	-1.06292
H	2.54254	2.68351	1.27476
H	-5.69553	1.26314	-0.76377
H	-2.38362	3.74741	0.49845
H	-5.86737	3.34579	0.07534
H	3.42484	0.84294	-2.23349
H	5.12005	1.10885	-1.77087
H	3.88912	2.32974	-1.38737
H	-0.44574	1.63794	-2.45352
H	-0.29064	3.2783	-1.7831
H	1.12459	2.19081	-1.80719
H	0.41214	0.09917	2.02326
H	-1.34499	0.08888	1.71219
H	-0.36069	-1.36436	1.36903
H	4.96863	-2.96422	1.52964
H	4.99002	-3.58009	-0.14725
H	6.51359	-3.23764	0.71034
H	6.32604	-0.44304	-1.75213
H	7.24513	-1.84252	-1.16379
H	5.72456	-2.08978	-2.04286
H	-5.28726	-0.81781	-1.92509
H	-3.61178	-1.30228	-2.31852
H	-3.38123	-2.04319	0.13026
H	-5.43095	-3.47269	-1.65101
H	-3.65684	-3.67153	-1.68964
H	-4.5821	-4.19756	-0.25705
C	-6.33223	-2.32736	2.62927
H	-6.06895	-2.98086	3.46658

H	-7.30666	-2.61333	2.21023
H	-6.38998	-1.28216	2.96199

2c			
C	6.23592	-0.63328	0.36443
C	6.38732	0.6402	-0.43753
C	5.30976	1.71383	-0.22567
C	3.89418	1.05912	-0.2183
C	3.71697	-0.29302	0.5328
C	4.85414	-1.24789	0.14013
C	2.7894	2.03309	0.19196
C	1.43687	1.42298	-0.00825
C	1.24178	0.09414	-0.06982
C	2.34953	-0.86942	0.14968
C	-0.05035	-0.54897	-0.50266
C	-0.33496	-1.81032	0.34712
C	0.7644	-2.81509	0.07162
C	2.12272	-2.20695	0.11935
C	-1.40094	0.18128	-0.4282
C	-2.41629	-0.90036	-0.31889
C	-1.77417	-2.10776	-0.00036
C	-3.79551	-0.85447	-0.57888
C	-4.49363	-2.0597	-0.51868
C	-3.84463	-3.26686	-0.21464
C	-2.46825	-3.30366	0.05872
O	-4.60015	-4.38059	-0.19754
O	0.58185	-4.00284	-0.06795
C	3.66058	-0.15174	2.07018
C	-0.29865	-1.49462	1.86379
C	0.09225	-0.90388	-2.01
O	-1.58827	1.37173	-0.52881
C	5.40458	2.71394	-1.38243
C	5.67475	2.45702	1.07523
O	7.31108	0.80705	-1.19311
H	0.57945	2.07957	-0.17173
C	-4.49288	0.43755	-0.90895
C	-4.65843	1.35401	0.30862
C	-5.45981	0.71832	1.44711
H	3.70118	0.806	-1.27741
C	-5.30351	2.65152	-0.13426
O	-5.71063	2.8893	-1.239
O	-5.37855	3.53502	0.87123
H	6.38101	-0.40055	1.4319
H	7.03735	-1.31745	0.05896
H	4.74414	-1.51605	-0.92374
H	4.78096	-2.18292	0.71345
H	2.91572	2.34656	1.24375
H	2.84613	2.95726	-0.4013
H	2.93093	-2.93213	0.218

H	-5.5644	-2.09451	-0.72526
H	-1.95267	-4.23761	0.29036
H	-4.0464	-5.14669	-0.00745
H	2.81633	0.47665	2.38807
H	4.5794	0.27315	2.49212
H	3.51545	-1.14738	2.51451
H	-0.99935	-0.67908	2.09409
H	-0.6133	-2.38389	2.42788
H	0.70457	-1.20192	2.19876
H	1.04226	-1.41934	-2.20186
H	-0.73205	-1.54493	-2.35182
H	0.08211	0.02672	-2.59371
H	5.1705	2.23021	-2.34155
H	4.71694	3.55754	-1.2352
H	6.42659	3.10584	-1.45439
H	5.6819	1.81439	1.96424
H	6.67722	2.89721	0.97123
H	4.96777	3.27924	1.25589
H	-3.9181	0.98556	-1.66916
H	-5.48428	0.23055	-1.33777
H	-3.65917	1.62972	0.68204
H	-6.46405	0.42721	1.10153
H	-4.95337	-0.1844	1.81604
H	-5.57041	1.42414	2.27984
C	-5.96423	4.78532	0.54666
H	-5.9504	5.38196	1.46416
H	-6.99534	4.64974	0.19276
H	-5.39083	5.28621	-0.24526

2d			
C	5.9667	1.14889	0.93361
C	6.22323	-0.33254	1.09766
C	5.48541	-1.26383	0.12474
C	3.99473	-0.82586	-0.0132
C	3.68638	0.69636	-0.10986
C	4.4674	1.44396	0.98128
C	3.25027	-1.58253	-1.11371
C	1.78532	-1.27597	-1.07537
C	1.28974	-0.1658	-0.50047
C	2.17511	0.88471	0.06162
C	-0.17549	0.0589	-0.2331
C	-0.5477	1.54067	-0.48295
C	0.21805	2.39312	0.50643
C	1.65416	2.01495	0.60236
C	-1.28167	-0.66776	-1.01178
C	-2.4685	0.22617	-0.90212
C	-2.05606	1.49366	-0.46476
C	-3.82646	-0.07753	-1.06396
C	-4.74852	0.9249	-0.76676
C	-4.33071	2.18535	-0.31266
C	-2.96951	2.48971	-0.16494
O	-5.29431	3.0771	-0.00811
O	-0.24607	3.35238	1.07956
C	3.99949	1.30963	-1.49259
C	-0.12823	1.99988	-1.90185
C	-0.45228	-0.35931	1.24007
O	-1.22253	-1.76561	-1.51467
C	5.5504	-2.68738	0.68755
C	6.28557	-1.23912	-1.19358
O	6.97887	-0.75596	1.93559
H	1.07988	-2.00699	-1.47687
C	-4.29042	-1.47828	-1.36753
C	-4.09979	-2.40389	-0.14338
C	-4.84482	-3.72339	-0.32781
H	3.52631	-1.13504	0.93958
C	-4.53793	-1.6612	1.10088
O	-3.80828	-1.26179	1.96839
O	-5.85977	-1.43609	1.10393
H	6.3882	1.47801	-0.03008
H	6.50745	1.67241	1.73201
H	4.07107	1.15098	1.96764
H	4.31322	2.52794	0.88486
H	3.66549	-1.3379	-2.10771
H	3.38428	-2.66768	-0.9959
H	2.28099	2.77246	1.07381
H	-5.82062	0.73654	-0.84448
H	-2.63302	3.46168	0.20102
H	-4.89058	3.89481	0.30515
H	3.40672	0.83513	-2.28758
H	5.05895	1.22772	-1.76399
H	3.7362	2.37742	-1.47808
H	-0.55802	1.32575	-2.65685
H	-0.51712	3.01124	-2.08582
H	0.96245	2.01421	-2.02265
H	0.23178	0.15647	1.92701
H	-1.49008	-0.15597	1.53886
H	-0.27994	-1.4407	1.33096
H	4.99883	-2.762	1.63562
H	5.13273	-3.41469	-0.02181
H	6.59313	-2.95989	0.89204
H	6.35412	-0.2437	-1.64924
H	7.30996	-1.58955	-0.99982
H	5.83583	-1.92089	-1.92939
H	-5.3562	-1.45988	-1.63879
H	-3.71867	-1.90574	-2.20275
H	-3.02574	-2.59115	-0.0117
H	-5.92574	-3.54468	-0.41065
H	-4.50059	-4.22267	-1.24433

H	-4.66943	-4.40315	0.51746
C	-6.34361	-0.62031	2.16076
H	-7.43048	-0.57133	2.04153
H	-5.90526	0.38594	2.0906
H	-6.08091	-1.0524	3.13528

2e			
C	6.23944	-0.63306	0.36383
C	6.39004	0.64164	-0.43625
C	5.31102	1.71377	-0.22405
C	3.89622	1.05743	-0.21827
C	3.72008	-0.29535	0.53175
C	4.85827	-1.2487	0.13836
C	2.78994	2.02978	0.19181
C	1.43816	1.41831	-0.0104
C	1.2447	0.08934	-0.07264
C	2.35341	-0.87299	0.14771
C	-0.04687	-0.55539	-0.50538
C	-0.33217	-1.81714	0.34489
C	0.76867	-2.82028	0.06521
C	2.12708	-2.21006	0.11728
C	-1.39565	0.17615	-0.43308
C	-2.41229	-0.90344	-0.3204
C	-1.77293	-2.11557	0.00107
C	-3.78816	-0.85097	-0.57726
C	-4.48939	-2.05733	-0.50977
C	-3.84623	-3.26553	-0.20069
C	-2.46801	-3.30783	0.0679
O	-4.51853	-4.42997	-0.14663
O	0.58948	-4.00611	-0.08534
C	3.66329	-0.15528	2.06918
C	-0.29485	-1.50178	1.86132
C	0.09501	-0.91205	-2.0125
O	-1.58343	1.36648	-0.53735
C	5.40551	2.71525	-1.37966
C	5.67438	2.45603	1.07783
O	7.3144	0.81082	-1.19064
H	0.5806	2.07442	-0.17458
C	-4.4862	0.44075	-0.90724
C	-4.64393	1.36248	0.30748
C	-5.42244	0.72593	1.46138
H	3.70429	0.80478	-1.27769
C	-5.31165	2.64914	-0.13334
O	-5.75532	2.86732	-1.22818
O	-5.36318	3.54543	0.86169
H	6.3839	-0.40157	1.43166
H	7.04168	-1.31604	0.0578
H	4.74878	-1.51575	-0.92583
H	4.78569	-2.18449	0.71049
H	2.91505	2.34225	1.24405
H	2.84644	2.95458	-0.40053
H	2.9353	-2.93525	0.21632
H	-5.5639	-2.06353	-0.71542
H	-1.9802	-4.25297	0.30177
H	-5.44729	-4.28423	-0.35959
H	2.81821	0.47196	2.38718
H	4.58149	0.27011	2.49199
H	3.51877	-1.15142	2.51256
H	-0.99653	-0.68727	2.09294
H	-0.60769	-2.39195	2.42488
H	0.70812	-1.20725	2.19539
H	1.045	-1.42772	-2.2037
H	-0.72879	-1.55483	-2.35246
H	0.08435	0.01763	-2.59772
H	5.17231	2.23233	-2.33941
H	4.71693	3.558	-1.2318
H	6.42719	3.10819	-1.4507
H	5.68198	1.81236	1.96605
H	6.6763	2.89773	0.97477
H	4.96609	3.27701	1.25912
H	-3.91562	0.98516	-1.6732
H	-5.48082	0.23493	-1.32977
H	-3.64248	1.65035	0.6648
H	-6.42826	0.42131	1.13167
H	-4.89968	-0.16733	1.83076
H	-5.53032	1.43689	2.29014
C	-5.96812	4.78726	0.53888
H	-5.93287	5.39505	1.44843
H	-7.00787	4.63908	0.21672
H	-5.42147	5.28264	-0.27507

2f			
C	-6.22677	0.71244	0.25805
C	-6.37912	-0.61407	-0.45191
C	-5.32175	-1.68179	-0.13603
C	-3.89794	-1.04581	-0.15027
C	-3.71611	0.35757	0.49996
C	-4.83353	1.29332	0.01508
C	-2.81459	-1.99951	0.35373
C	-1.45036	-1.42275	0.13608
C	-1.23603	-0.10479	-0.02196
C	-2.33451	0.88606	0.09782
C	0.07299	0.48484	-0.47939
C	0.35858	1.8105	0.2659
C	-0.72317	2.80062	-0.11205
C	-2.0899	2.21387	-0.03832
C	1.41148	-0.25234	-0.31241
C	2.43895	0.82251	-0.27585
C	1.80716	2.06132	-0.08011
C	3.82233	0.73699	-0.49926
C	4.53486	1.93438	-0.53898
C	3.89597	3.17194	-0.3635
C	2.51583	3.2496	-0.11803
O	4.66498	4.27362	-0.44019
O	-0.52305	3.9704	-0.34702
C	-3.6902	0.33523	2.04472
C	0.29268	1.62374	1.80267
C	-0.03479	0.71305	-2.01363
O	1.58514	-1.44889	-0.3047
C	-5.40926	-2.76846	-1.21223
C	-5.72233	-2.31483	1.21155
O	-7.28873	-0.82441	-1.21381
H	-0.59869	-2.10013	0.04233
C	4.51448	-0.58852	-0.67421
C	4.6453	-1.36853	0.65038
C	5.30616	-0.57438	1.76818
H	-3.68223	-0.87484	-1.22135
C	5.39388	-2.6677	0.41985
O	6.27874	-3.09066	1.11355
O	4.94817	-3.32144	-0.66123
H	-6.39284	0.55875	1.33658
H	-7.01375	1.3821	-0.11062
H	-4.70083	1.47984	-1.06355
H	-4.7584	2.26768	0.5183
H	-2.96455	-2.23187	1.42307
H	-2.87363	-2.96489	-0.16961
H	-2.89061	2.95356	-0.01441
H	5.61056	1.9384	-0.72144
H	2.00916	4.20702	0.01823
H	4.12132	5.06005	-0.31508
H	-2.86787	-0.28561	2.42796
H	-4.62712	-0.03413	2.47977
H	-3.52929	1.35873	2.41383
H	0.97866	0.82247	2.11363
H	0.60929	2.5535	2.29593
H	-0.72002	1.37147	2.14221
H	-0.97173	1.22579	-2.26723
H	0.80653	1.31043	-2.39117
H	-0.02948	-0.26347	-2.51705
H	-5.14497	-2.36621	-2.2007
H	-4.74177	-3.61025	-0.98324
H	-6.43679	-3.14697	-1.27692
H	-5.73377	-1.60377	2.04644
H	-6.7303	-2.74595	1.12358
H	-5.03363	-3.1315	1.47103
H	3.94765	-1.21389	-1.37714
H	5.5167	-0.41828	-1.09773

H	3.62405	-1.66751	0.94215
H	6.30348	-0.22536	1.45965
H	4.70016	0.30196	2.03511
H	5.43823	-1.20247	2.65781
C	5.57836	-4.56125	-0.9377
H	5.44116	-5.26009	-0.10134
H	6.65611	-4.42015	-1.09671
H	5.10546	-4.95274	-1.84375

2g

C	-6.29397	0.62908	-0.38628
C	-6.35572	-0.8347	-0.76023
C	-5.37592	-1.76598	-0.03127
C	-3.95684	-1.12168	0.01791
C	-3.85513	0.40357	0.3139
C	-4.87105	1.16323	-0.5515
C	-2.98261	-1.89217	0.90959
C	-1.59171	-1.35411	0.77512
C	-1.33269	-0.1145	0.32263
C	-2.42083	0.84368	0.00295
C	0.04111	0.36651	-0.06722
C	0.22979	1.85202	0.3171
C	-0.76572	2.66915	-0.47945
C	-2.13716	2.09017	-0.45166
C	1.32718	-0.26091	0.49566
C	2.35739	0.81108	0.37521
C	1.7173	2.03911	0.13637
C	3.75601	0.71772	0.40206
C	4.48029	1.8849	0.15737
C	3.83687	3.10295	-0.10375
C	2.43575	3.19621	-0.10685
O	4.61862	4.17275	-0.34228
O	-0.51363	3.73123	-1.00108
C	-4.06318	0.76776	1.80058
C	-0.07341	2.09236	1.81743
C	0.17636	0.16383	-1.60417
O	1.47106	-1.40053	0.86963
C	-5.31281	-3.08659	-0.8048
C	-5.99168	-2.05389	1.35304
O	-7.14118	-1.24876	-1.57507
H	-0.74688	-2.01071	0.99443
C	4.47745	-0.58804	0.62096
C	4.90143	-1.24709	-0.70984
C	3.72893	-1.60585	-1.61211
H	-3.57125	-1.22062	-1.01397
C	5.74385	-2.47868	-0.43153
O	5.53967	-3.57687	-0.87053
O	6.78254	-2.2018	0.37109
H	-6.62423	0.74278	0.65883
H	-7.00524	1.16892	-1.02365
H	-4.57345	1.08046	-1.60998
H	-4.85848	2.23341	-0.30028
H	-3.305	-1.85657	1.96545
H	-2.97139	-2.95867	0.6406
H	-2.91926	2.78633	-0.75571
H	5.57195	1.87492	0.15929
H	1.92301	4.13921	-0.30635
H	4.06844	4.94809	-0.50282
H	-3.31642	0.28221	2.44462
H	-5.06016	0.49466	2.16783
H	-3.94672	1.85484	1.92128
H	0.55545	1.43746	2.43771
H	0.16291	3.1347	2.07429
H	-1.12685	1.89902	2.05771
H	-0.72166	0.52197	-2.12422
H	1.04902	0.69787	-2.00556
H	0.29038	-0.90901	-1.8134
H	-4.89428	-2.93428	-1.80995
H	-4.69962	-3.82864	-0.27587
H	-6.3232	-3.49509	-0.92929
H	-6.11344	-1.15931	1.97636
H	-6.98448	-2.50842	1.22095
H	-5.36948	-2.77117	1.90708
H	5.37983	-0.40164	1.2184
H	3.83029	-1.28266	1.17326
H	5.5731	-0.54048	-1.22913
H	3.04181	-2.28759	-1.09115
H	3.17451	-0.70209	-1.90153
H	4.08103	-2.10794	-2.52218
C	7.63717	-3.29393	0.671
H	8.42098	-2.90232	1.32673
H	8.07473	-3.70502	-0.24897
H	7.07893	-4.09406	1.17562

4. Biological assays

Table S9. Cytotoxicity activities of compounds **1** and **2**

Compound	Cell inhibition rates (%)[a]				
	HL-60	A-549	SMMC-7721	MCF-7	SW480
**1**	8.31 ± 2.58	3.95 ± 1.36	8.35 ± 0.91	10.43 ± 2.27	17.78 ± 1.77
**2**	11.62 ± 2.01	2.73 ± 0.51	7.68 ± 0.65	5.77 ± 0.91	13.49 ± 3.01
**DDP**[b]	93.05	82.50	81.4	67.85	70.8
**Taxol**[c]	90.33	58.14	83.93	56.61	60.04

[a] Compounds **1** and **2** were tested at the concentration of 40 μM.

[b] DDP was used as positive control at the concentration of 40 μM.

[c] Taxol was used as positive control at the concentration of 5μM against cell line SMMC-7721 and at the concentration of 0.008 μM against the rest four cell lines.

Table S10. Inhibition rates of compounds **1** and **2** against BACE1

Compound	Inhibition rates (%)[a]	IC_{50}
**1**	25.58	–
**2**	44.67	–
**LY2811376**[b]	–	0.6 μM

[a] Compounds **1** and **2** were tested at the concentration of 40 μM.

[b] LY2811376 was used as positive control.

Table S11. Inhibition rates of compounds **1** and **2** against α-glucosidase

Compound	Inhibition rates (%)[a]
**1**	−7.45 ± 0.42
**2**	1.43 ± 1.92
**Quercetin**[b]	54.49 ± 2.23

[a] Compounds **1** and **2** were tested at the concentration of 50 μM.

[b] Quercetin was used as positive control at the concentration of 10 μM.

Table S12. Inhibition rates of compounds **1** and **2** against PTP1B

Compound	Inhibition rates (%)[a]
**1**	−5.04 ± 3.07
**2**	5.59 ± 2.44
**Suramin**[b]	96.09 ± 1.16

[a] Compounds **1** and **2** were tested at the concentration of 50 μM.

[b] Suramin was used as positive control at the concentration of 20 μM.
